# Picrasidine S Induces cGAS‐Mediated Cellular Immune Response as a Novel Vaccine Adjuvant

**DOI:** 10.1002/advs.202310108

**Published:** 2024-06-20

**Authors:** Xiaofan Ding, Mengxue Sun, Fusheng Guo, Xinmin Qian, Haoyu Yuan, Wenjiao Lou, Qixuan Wang, Xiaoguang Lei, Wenwen Zeng

**Affiliations:** ^1^ Institute for Immunology and School of Basic Medical Sciences and Beijing Key Laboratory for Immunological Research on Chronic Diseases Tsinghua University Beijing 100084 China; ^2^ Beijing National Laboratory for Molecular Sciences Key Laboratory of Bioorganic Chemistry and Molecular Engineering of Ministry of Education College of Chemistry and Molecular Engineering Peking University Beijing 100871 China; ^3^ Peking‐Tsinghua Center for Life Sciences Academy for Advanced Interdisciplinary Studies Peking University Beijing 100871 China; ^4^ Institute of Cancer Research Shen Zhen Bay Laboratory Shen Zhen 518107 China; ^5^ SXMU‐Tsinghua Collaborative Innovation Center for Frontier Medicine Taiyuan 030001 China; ^6^ Tsinghua‐Peking Center for Life Sciences Beijing 100084 China

**Keywords:** adjuvant, cancer prevention, cellular immune response, cGAS, type I interferon

## Abstract

New adjuvants that trigger cellular immune responses are urgently needed for the effective development of cancer and virus vaccines. Motivated by recent discoveries that show activation of type I interferon (IFN‐I) signaling boosts T cell immunity, this study proposes that targeting this pathway can be a strategic approach to identify novel vaccine adjuvants. Consequently, a comprehensive chemical screening of 6,800 small molecules is performed, which results in the discovery of the natural compound picrasidine S (PS) as an IFN‐I inducer. Further analysis reveals that PS acts as a powerful adjuvant, significantly enhancing both humoral and cellular immune responses. At the molecular level, PS initiates the activation of the cGAS‐IFN‐I pathway, leading to an enhanced T cell response. PS vaccination notably increases the population of CD8+ central memory (T_CM_)‐like cells and boosts the CD8+ T cell‐mediated anti‐tumor immune response. Thus, this study identifies PS as a promising candidate for developing vaccine adjuvants in cancer prevention.

## Graphical abstract

This study performs a comprehensive chemical screening of 6800 small molecules and identifies picrasidine S (PS) as a potent IFN‐I inducer. PS acts as a powerful adjuvant, significantly enhancing both humoral and cellular immune responses. It activates the cGAS‐IFN‐I pathway, leading to an increase in CD8+ central memory (T_CM_)‐like cells and improves CD8+ T cell‐mediated anti‐tumor immunity.

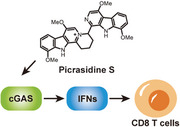

## 1 Introduction

Vaccination is among the most effective methods to prevent diseases and safeguard public health.^[^ ^1^, ^2^ ^]^ Adjuvants, typically non‐antigenic substances, enhance antigen‐specific immune responses.^[^ ^3^, ^4^, ^5^ ^]^ However, the development of new vaccine adjuvants has been one of the slowest aspects of preventive healthcare innovation.^[^ ^6^ ^]^ To date, only a few adjuvants have received approval from the U.S. Food and Drug Administration (FDA) for use in human vaccines. Aluminium hydroxide, discovered in the 1920s, was the first adjuvant and remained predominant in licensed products for over 70 years. In the past 30 years, adjuvants such as MF59, AS01, AS03, AS04, cytosine phosphoguanosine (CpG) 1018, and MatrixM have been approved for human use.^[^ ^4^ ^]^

Most current vaccines primarily confer protection through humoral immunity.^[^ ^7^, ^8^ ^]^ Alum is the most commonly used adjuvant in licensed vaccines, including those for hepatitis A, hepatitis B, pertussis, and human papilloma virus.^[^ ^9^ ^]^ These vaccines owe their effectiveness to the ability of alum to elicit strong T helper 2 (T_H_2) cell responses, which generate high antibody levels. However, alum adjuvants are less effective at inducing T helper 1 (T_H_1) or cytotoxic T lymphocyte (CTL) immune responses, making them suboptimal for generating the cellular immune responses crucial for defending against intracellular pathogens and tumors.^[^ ^10^, ^11^, ^12^ ^]^ Additionally, alum can provoke unwanted IgE antibodies and cause granulomas when injected subcutaneously, intradermally, or administered repeatedly.^[^ ^12^, ^13^ ^]^ Consequently, there is an urgent need for new vaccine adjuvants that enhance cellular immune responses.

From 1990 to 2010, extensive research was conducted to identify tumor‐associated antigens and integrate them with various adjuvants and delivery systems to enhance their immunogenicity and clinical efficacy.^[^ ^14^, ^15^ ^]^ Despite these efforts, the overall impact remained limited, primarily due to the inability to stimulate the CTL immune response. In the quest for an effective vaccine design strategy, numerous studies have highlighted that the immune system has evolved distinct response mechanisms to combat different pathogens.^[^ ^16^, ^17^, ^18^ ^]^ The activation of the innate immune response is the critical initial event that significantly influences the outcome of the adaptive immune response, whether it leads to the production of high‐affinity antibodies or a cellular immune response mediated by cytotoxic T cells7.^[^ ^7^, ^19^, ^20^, ^21^, ^22^ ^]^ Although controlling tumor progression or viral infection depends largely on the latter, only recent developments have shown that the production of type I interferon (IFN‐I) by dendritic cells (DCs) or nonhematopoietic stromal cells that do not present the antigen can greatly affect T_H_1^[^ ^23^, ^24^ ^]^ and CD8+ T cell immunity.^[^ ^7^, ^25^, ^26^, ^27^, ^28^ ^]^ Consequently, modulating the early innate immune response has emerged as a viable approach to enhance adaptive immunity during immunization.

In this study, we hypothesized that targeting IFN‐I could be an effective strategy to enhance T cell immunity. Through extensive chemical screening of a small molecule library (≈6800 compounds, including FDA‐approved drugs, natural products, and known tool compounds), we found that the natural product picrasidine S (PS) is a potent inducer of IFN‐I and an effective vaccine adjuvant. Compared to alum adjuvant, PS demonstrates significantly enhanced adjuvant activity in cellular immune responses. We show that PS modulates immune reactions through the cGAS‐IFN‐I pathway. Additionally, PS vaccination notably increases the CD8+ central memory (T_CM_)‐like cells and mediates robust protective CD8+ T cell‐mediated anti‐tumor immunity. These findings suggest a promising new candidate for developing vaccine adjuvants and cancer prevention strategies.

## 2 Results

### 2.1 PS Induces IFN‐β Production

**Figure 1:**
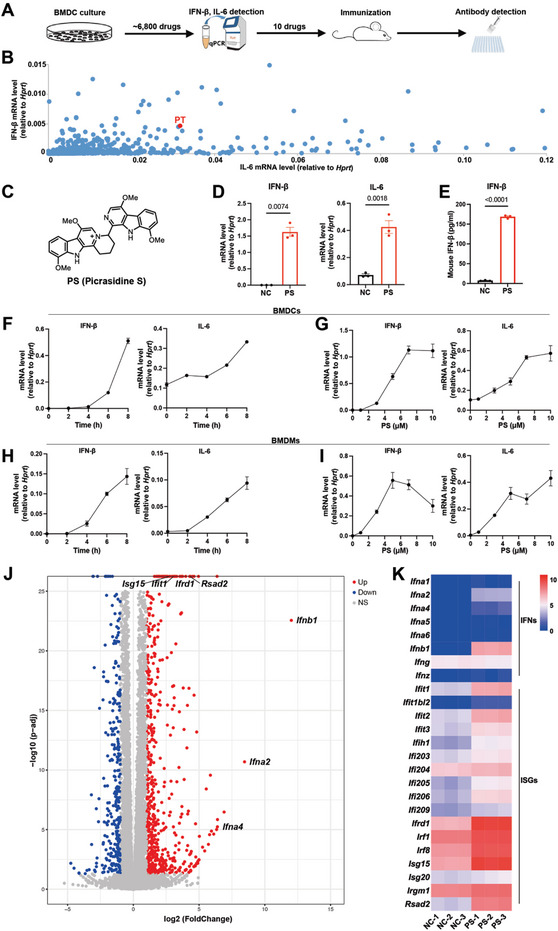
PS induces IFN‐β production. A) Workflow of drug screening. B) 10 compounds were pooled in the first round of screening, and expression levels of IFN‐β and IL‐6 in the bone marrow‐derived dendritic cells (BMDCs) post drug stimulation were determined by the qPCR analysis. C) The chemical structure of picrasidine S (PS). D) Expression levels of IFN‐β and IL‐6 in BMDCs post PS stimulation were determined by the qPCR analysis (n = 3). E) Protein level of IFN‐β in BMDCs post PS stimulation was determined by the ELISA analysis (n = 3). F,H) Expression levels of IFN‐β and IL‐6 in BMDCs (F) or BMDMs (H) at indicated time points post 10 µm PS stimulation were determined by the qPCR analysis (n = 3). G,I) Expression levels of IFN‐β and IL‐6 in BMDCs (G) or BMDMs (I) at varied PS concentrations for 8 h were determined by the qPCR analysis (n = 3). J) Volcano plot displaying genes that were highly expressed in BMDCs post PS stimulation by RNA‐Seq analysis (n = 3). K) Heatmap showing the feature genes related to IFN‐I in BMDCs post PS stimulation by RNA‐Seq analysis (n = 3). Data are presented as mean ± SEM. *P* values were calculated by Student's t test (D,E).

To explore pharmacological approaches for activating IFN‐I signaling, we began by identifying compounds that could stimulate the production of IFN‐β, which is notably higher in macrophages and dendritic cells compared to the production of IFN‐α.^[^ ^29^ ^]^ We conducted a comprehensive screening of 6800 compounds, including FDA‐approved drugs, natural products, and established tool compounds. Initially, 10 compounds were grouped together in the first screening phase and subsequently separated in the second phase to test their effects on bone marrow‐derived dendritic cells (BMDCs). We also evaluated the transcript levels of interleukin‐6 (IL‐6) to filter out compounds that might cause an excessive inflammatory response (**Figure** **1**A,B).

Out of the 6800 compounds screened, 10 were identified that significantly induced IFN‐β and modestly induced IL‐6. To assess their activity in vivo, we immunized C57BL/6 mice with 4‐hydroxy‐3‐nitrophenyl hapten conjugated to keyhole limpet hemocyanin (NP‐KLH) along with various drugs (structures depicted in Figure S1A, Supporting Information). Subsequently, we measured NP‐specific antibody titers after 14 days. Among the tested compounds, picrasidine T (PT, 6‐K15) demonstrated a robust humoral immune response (Figure S1B, Supporting Information), with significantly elevated levels of NP‐specific IgG, IgG1, IgG2b, IgG2C, and IgG3 titers compared to NP‐KLH alone.

We then evaluated the activity of 10 PT derivatives (structures in Figure S2A, Supporting Information) in inducing IFN‐β and IL‐6 in BMDCs. Picrasidine S (PS, structure in Figure 1C) showed the highest IFN‐β induction activity (Figure S2B, Supporting Information). We confirmed IFN‐β and IL‐6 induction by PS using qPCR (Figure 1D) and verified increased IFN‐β protein levels via ELISA (Figure 1E). PS induced IFN‐β in a dose‐ and time‐dependent manner in both BMDCs and bone marrow‐derived dendritic cells (BMDMs) (Figure 1F–I). The EC_50_ of PS in BMDCs was determined through qPCR (Figure S2C, Supporting Information), showing 5.94 and 5.36 µM for IFN‐β and IL‐6 induction, respectively. RNA‐seq analysis of PS‐treated BMDCs indicated robust production of IFN‐β, various IFN‐αs, and interferon‐stimulated genes (ISGs) (Figure 1J,K), suggesting activation of the IFN‐I signaling pathway. Notably, IFN‐β expression was highest among the IFNs following PS treatment (Figure 1K). These findings confirm PS as a potent IFN‐β inducer.

### 2.2 PS Enhances Humoral Immunity in Vaccination

**Figure 2:**
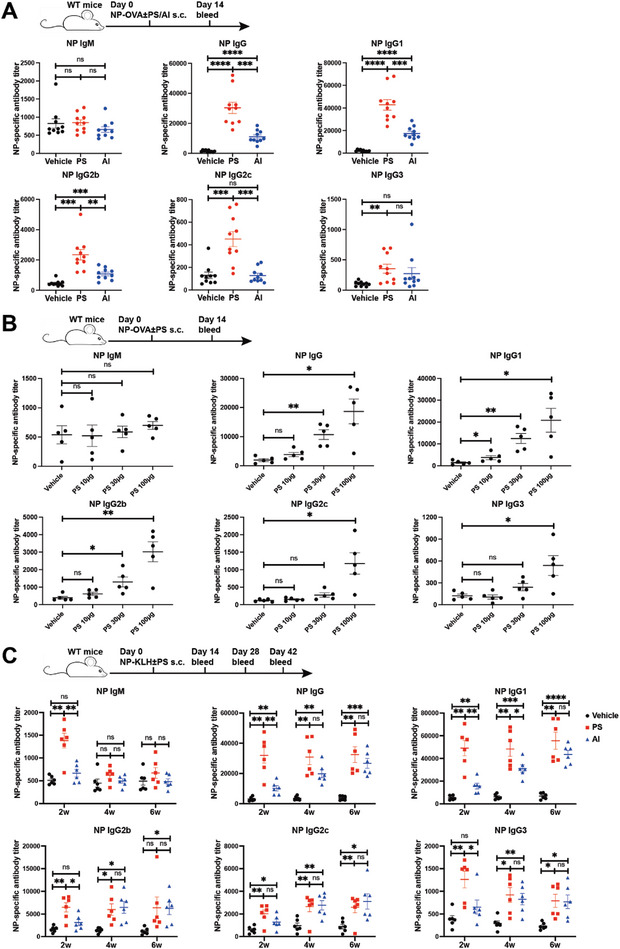
PS enhances humoral immunity in vaccination. A) C57BL/6 mice were immunized with 50 µg NP‐OVA, 50 µg NP‐OVA+100 µg PS or 50 µg NP‐OVA+25 µL Alum on day 0. Serum NP‐specific antibody titers in IgM, IgG, IgG1, IgG2b, IgG2C, and IgG3 isotypes on day 14 were examined by ELISA (n = 10). B) C57BL/6 mice were immunized with 50 µg NP‐OVA or 50 µg NP‐OVA in combination with indicated dose of PS on day 0. Serum NP‐specific antibody titers in IgM, IgG, IgG1, IgG2b, IgG2C, and IgG3 isotypes on day 14 were examined by ELISA (n = 5). C) C57BL/6 mice were immunized with 50 µg NP‐KLH, 50 µg NP‐KLH+150 µg PS, or 50 µg NP‐KLH+25 µL Alum on day 0. Serum NP‐specific antibody titers in IgM, IgG, IgG1, IgG2b, IgG2C, and IgG3 isotypes on day 14, day 28, and day 42 were examined by ELISA (n = 6). Data are presented as mean ± SEM. *P* values were calculated by Student's *t* test (A, B, C). ns (nonsignificant) *p* > 0.05, ^*^ *p* < 0.05, ^**^ *p* < 0.01, ^***^ *p* < 0.001, ^****^ *p* < 0.0001.

To assess PS as a potential vaccine adjuvant, we conducted a comparative analysis of the humoral responses induced by PS and alum, the most commonly used adjuvant in clinical settings. C57BL/6 mice were immunized with 4‐hydroxy‐3‐nitrophenyl hapten conjugated to ovalbumin (NP‐OVA) alongside either PS or alum. The results indicated that PS generated the highest titers of NP‐specific IgG, IgG1, IgG2b, IgG2c, and IgG3 antibodies (**Figure** **2**A). Additionally, we evaluated various doses of PS (10, 30, and 100 µg per animal) and observed a dose‐dependent increase in the humoral response (Figure 2B). Notably, PS significantly enhanced antibody titers against the NP‐KLH antigen, outperforming alum in efficacy (Figure 2C). These findings suggest that PS boosts humoral immunity in vaccinations and is more effective than alum as an adjuvant.

### 2.3 PS Enhances Cellular Immunity and Anti‐Tumor Response in Vaccination

**Figure 3:**
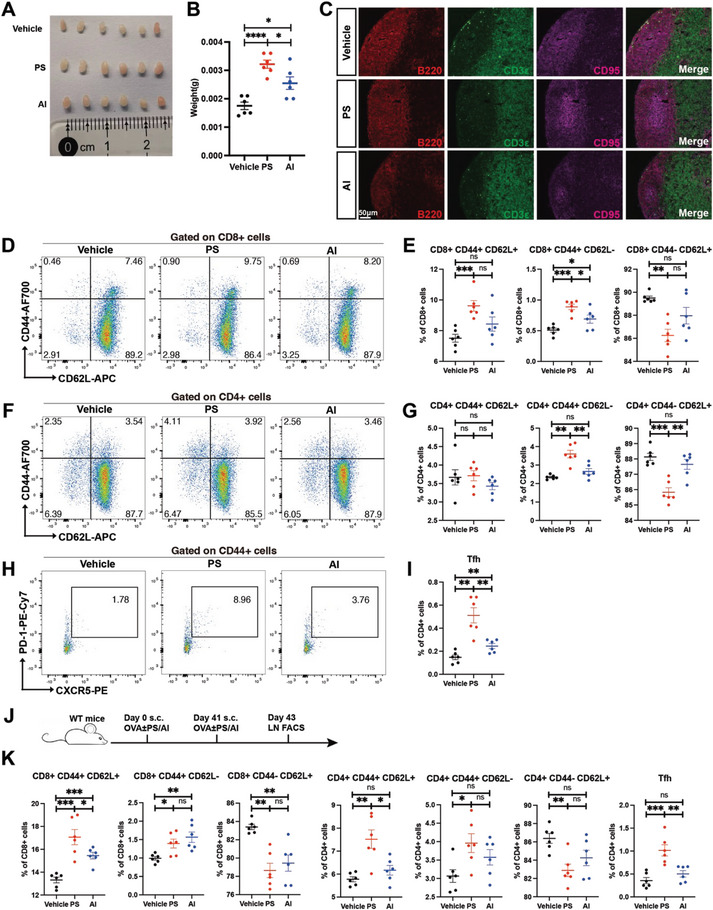
PS enhances cellular immunity in vaccination. A–C) C57BL/6 mice were immunized on day 0 with 50 µg OVA, 50 µg OVA+100 µg PS, or 50 µg OVA+25 µL alum. (A,B) On day 6, subcutaneous lymph nodes were removed and organ weights were recorded (n = 6). (C) Immunofluorescence analysis of subcutaneous lymph node sections on day 9. Antibodies detecting B220, CD3ε and CD95 (which marks germinal center B cells) were used (n = 5). Scale bars: 50 µm. D–I) C57BL/6 mice were immunized on day 0 with 50 µg NP‐OVA, 50 µg NP‐OVA+100 µg PS or 50 µg NP‐OVA+25 µL alum. On day 9, subcutaneous lymph nodes were removed for flow‐cytometry analysis (n = 6). (D–G) The percentage of standard subsets of CD8+ and CD4+ T cells, including central memory (T_CM_, CD44+ CD62L+), effector memory (T_EM_, CD44+ CD62L‐), and naïve (CD44‐ CD62L+). H,I) The percentage of T follicular helper cells (Tfh, CD44+ CXCR5+ PD‐1+). J,K) C57BL/6 mice were immunized with 50 µg OVA, 50 µg OVA+100 µg PS, or 50 µg OVA+25 µL alum on day 0 and day 41. On day 43, subcutaneous lymph nodes were removed for flow‐cytometry analysis (n = 6). (K) The percentage of standard subsets of CD8+ and CD4+ T cells. Data are presented as mean ± SEM. *P* values were calculated by Student's *t* test (B,E,G,I,K). ns (nonsignificant) *p* > 0.05, ^*^ *p* < 0.05, ^**^ *p* < 0.01, ^***^ *p* < 0.001, ^****^ *p* < 0.0001.

To assess whether PS could boost the cellular immune response of vaccines, we examined subcutaneous lymph nodes after mice were immunized with OVA combined with PS or alum. The findings indicated that the lymph nodes in the PS‐treated group were significantly larger than those in the control and alum adjuvant group (**Figure** **3**A,B). Additionally, the development of germinal centers in the lymph nodes was more evident, labeled by co‐immunostainig of CD95, B220, and CD3e (Figure 3C). Flow cytometry analysis showed an increased presence of CD8+ CD44+ CD62L+ T_CM_‐like and CD8+ CD44+ CD62L‐ T_EM_‐like cells in the lymph nodes of the PS‐treated group, accompanied by a reduction in CD8+ CD44‐ CD62L+ naive T cells (Figure 3D,E). A similar pattern was observed for CD4+ T cells, with an increase in CD4+ CD44+ CD62L‐ T_EM_‐like cells and a decrease in CD4+ CD44‐ CD62L+ naive T cells (Figure 3F,G). Moreover, CD4+ CD44+ CXCR5+ PD‐1+ Tfh cells, crucial for supporting antibody production, also increased in the lymph nodes of the PS‐treated group (Figure 3H,I), aligning with the elevated antibody titers. To further confirm the impact of PS immunization on T cells, mice underwent a secondary immunization 41 days after the initial dose, and T cell subsets in the lymph nodes were analyzed two days later (Figure 3J). There was a notable increase in both CD8+ and CD4+ T_CM_‐like and T_EM_‐like cells, as well as Tfh cells (Figure 3K), consistent with the primary immunization results. Additionally, when DMXAA, a previously characterized vaccine adjuvant that activates the stimulator of interferon genes (STING) pathway to produce IFN‐I^[^ ^30^ ^]^ was used as a control, comparable effects on lymph node response, germinal center formation and immune reactions were observed (Figure S3, Supporting Information). These findings demonstrate that PS immunization enhances the cellular immune response, particularly by promoting the formation of CD8+ T_CM_‐like cells.

**Figure 4:**
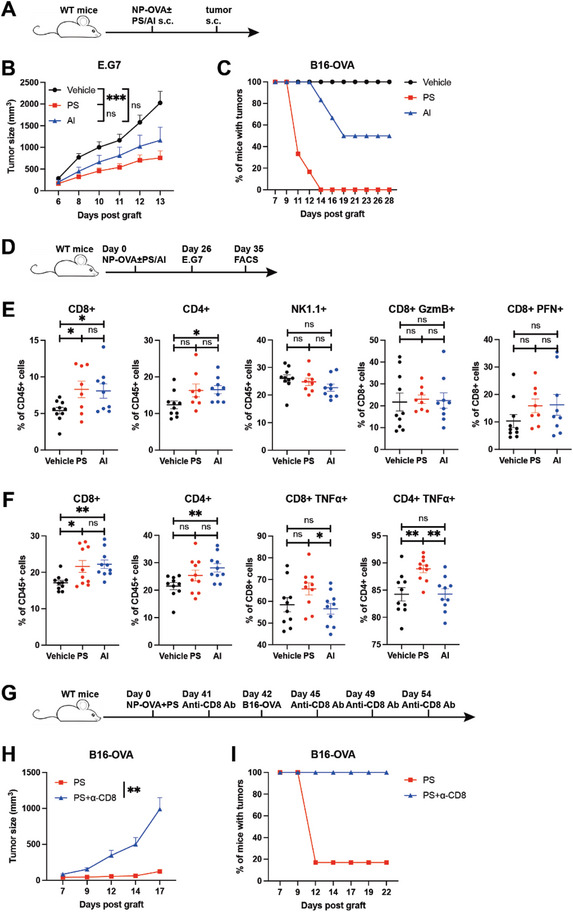
PS promotes anti‐tumor immunity in a CD8+ T cell‐dependent manner. A–C) C57BL/6 mice were immunized with 50 µg NP‐OVA, 50 µg NP‐OVA+100 µg PS or 50 µg NP‐OVA+25 µL alum. (B) Mice were challenged s.c. with 5 × 10^6^ of E.G7 cells 38 days post immunization. Tumor growth was monitored over time (n = 10). (C) Mice were challenged s.c. with 2 × 10^6^ of B16‐OVA cells 23 days post immunization. Tumor incidence was monitored over time (n = 6). D–F) C57BL/6 mice were immunized on day 0 with 50 µg NP‐OVA, 50 µg NP‐OVA+100 µg PS or 50 µg NP‐OVA+25 µL alum. 26 days post immunization, mice were challenged s.c. with 3 × 10^6^ of E.G7 cells. On day 35, tumors and draining lymph nodes were removed for flow‐cytometry analysis. (E) Percentages of CD8+, CD4+, NK1.1+ TILs and CD8+ TILs expressing GzmB or PFN in tumors (n = 10+8+9). (F) Percentages of CD8+, CD4+ T cells, and T cells producing TNFα induced by PMA and ionomycin in draining lymph nodes(n = 10). G–I) C57BL/6 mice were immunized on day 0 with 50 µg NP‐OVA+100 µg PS. 42 days post immunization, mice were challenged s.c. with 3 × 10^6^ of B16‐OVA cells. CD8‐depleting antibody was injected at the indicated time points. Tumor growth (H) and tumor incidence (I) were monitored over time (n = 6). Data are presented as mean ± SEM. *P* values were calculated by Student's t test (E and F) and two‐way ANOVA (B,H). ns (nonsignificant) *p* > 0.05, ^*^ *p* < 0.05, ^**^ *p* < 0.01, ^***^ *p* < 0.001.

Next, we assessed the prophylactic efficacy of PS in various tumor models. Initially, wild‐type mice were immunized with NP‐OVA combined with either PS or alum adjuvant. After immunization, these mice were injected with tumor cells expressing the OVA antigen (E.G7 or B16‐OVA) (**Figure** **4**A), and tumor growth was monitored over time. The results indicated that the PS‐treated group exhibited smaller tumor sizes (Figure 4B) and lower tumor incidence (Figure 4C) compared to the control and alum adjuvant groups. Analysis of tumor‐infiltrating lymphocytes (TILs) revealed increased CD8+ TILs in the PS‐treated group, whereas CD4+ and NK1.1+ TILs showed no significant changes. Additionally, there was no difference in the expression of granzyme B (GzmB) or perforin (PFN) in CD8+ TILs (Figure 4E). Furthermore, examination of tumor‐draining lymph nodes showed an increase in CD8+ T cells in the PS‐treated group, with more CD4+ T cells expressing TNFα (Figure 4F). Other immune cell subsets in tumors (Figure S4B,C, Supporting Information) and tumor‐draining lymph nodes (Figure S4D, Supporting Information) were also analyzed, including NK1.1+, CD11c+, and CD11b+ cells, but no differences were observed, highlighting the crucial role of CD8+ T cells in the PS‐induced anti‐tumor immune response.

To further confirm the role of CD8+ T cells in the PS‐induced anti‐tumor response, wild‐type mice were immunized with NP‐OVA along with PS and subsequently received anti‐CD8 antibody injections before and after B16‐OVA inoculation (Figure 4G). The administration of the anti‐CD8 antibody successfully inhibited the PS‐mediated anti‐tumor response (Figure 4H,I). Conversely, the administration of anti‐CD4 antibody did not affect the PS‐mediated anti‐tumor response (Figure S4E–G, Supporting Information). Collectively, these findings reinforce that the anti‐tumor immune responses induced by PS immunization are reliant on CD8+ T cells.

PS‐mediated vaccination was well‐tolerated in C57BL/6 mice, as indicated by minimal effects on body weight (Figure S5B, Supporting Information), survival (Figure S5C, Supporting Information), and gross organ anatomy (Figure S5D–F, Supporting Information). The pharmacokinetic parameters of PS were evaluated using LC‐MS/MS following a single intravenous dose to assess its plasma exposure and stability in vivo. The results revealed favorable profiles, including a reasonable half‐life of 5.5 h, an ideal maximum plasma concentration of 8423 ng mL^−1^, and an acceptable area under the curve of 3656 ng mL^−1^*h (Figure S5G, Supporting Information). In summary, our findings confirm that PS is an effective adjuvant, promoting balanced humoral and cellular immune responses with desirable safety and pharmacokinetic characteristics.

### 2.4 PS Activates the cGAS‐IFN‐I Pathway

**Figure 5:**
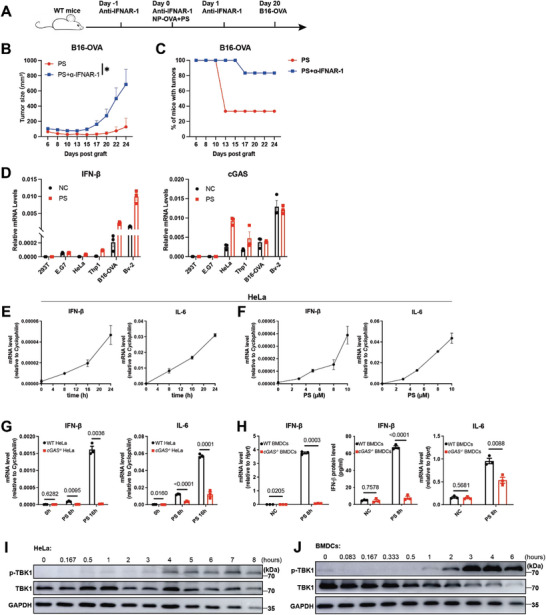
PS activates the cGAS‐IFN‐I pathway. A–C) C57BL/6 mice were immunized on day 0 with 50 µg NP‐OVA+100 µg PS. 20 days post immunization, mice were challenged s.c. with 3 × 10^6^ of B16‐OVA cells. Anti‐IFNAR‐1 antibody was injected at the indicated time points. Tumor growth (B) and tumor incidence (C) were monitored over time (n = 6). D) Expression levels of IFN‐β and cGAS in different cell types post PS stimulation were determined by the qPCR analysis (n = 3). E) Expression levels of IFN‐β and IL‐6 in HeLa cells at indicated time points post 10 µm PS stimulation were determined by the qPCR analysis (n = 3). F) Expression levels of IFN‐β and IL‐6 in HeLa cells at varied PS concentrations for 16 h were determined by the qPCR analysis (n = 3). G) Expression levels of IFN‐β and IL‐6 in WT and cGAS‐deficient (*cGAS^−/−^*) HeLa cells post PS stimulation were determined by the qPCR analysis (n = 3). H) Expression and protein levels of IFN‐β and IL‐6 in WT and cGAS^−/−^ BMDCs post PS stimulation were determined by the qPCR and ELISA analysis (n = 3). (I,J) Immunoblot analysis of HeLa cells I) and BMDCs J) post PS stimulation. Data are presented as mean ± SEM. *P* values were calculated by Student's *t* test (G,H) and two‐way ANOVA (B). ^*^ *p* < 0.05.

Our results demonstrated that PS induced the expression of IFN‐β in vitro (Figure 1D–K). To determine if the PS‐induced immune response relies on IFN‐I in vivo, wild‐type mice were pre‐treated with anti‐IFNAR‐1 antibody before and after immunization with NP‐OVA combined with PS, followed by inoculation with B16‐OVA tumor cells (**Figure** **5**A). The administration of anti‐IFNAR‐1 antibody effectively inhibited the PS‐mediated anti‐tumor response (Figure 5B,C). Additionally, we immunized IFNα/β‐receptor (IFNAR)‐knockout (IFNα/βR^−/−^) mice with OVA and PS and examined subcutaneous lymph nodes. The findings indicated that the size and weight of lymph nodes in IFNα/βR^−/−^ mice were significantly reduced compared to the control group (Figure S6A,B, Supporting Information), and the development of germinal centers in the lymph nodes was substantially reduced (Figure S6C, Supporting Information). Flow cytometry analysis showed a reduction in CD8+ T cells, CD4+ T cells, T_CM_ and T_EM_‐like cells, NK1.1+ cells, and Tfh cells in the lymph nodes of IFNα/βR^−/−^ mice (Figure S6D, Supporting Information). Moreover, when MC38‐OVA tumor cells were inoculated into OVA/PS immunized mice (Figure S6E, Supporting Information), IFNα/βR^−/−^ mice exhibited a diminished anti‐tumor response (Figure S6F, Supporting Information). Conversely, PS‐induced antibody titers were lower in IFNα/βR^−/−^ mice (Figure S6G, Supporting Information). These findings collectively confirm that IFN‐I plays a crucial role in the PS‐induced cellular and humoral immune responses.

To investigate the molecular mechanisms behind PS‐induced IFN‐I, we analyzed IFN‐β production in various cell lines following PS treatment and observed that PS robustly triggers IFN‐β production in cells expressing cGAS (Figure 5D). Further studies on the impact of PS on HeLa cells, varying exposure times and concentrations, demonstrated that IFN‐β expression is both time and dose‐dependent (Figure 5E,F). In cGAS‐deficient HeLa and BMDC cells, the increase in IFN‐β levels induced by PS was significantly reduced (Figure 5G,H). Additional investigations into the cGAS downstream signaling protein, p‐TBK1, revealed a marked elevation post‐PS stimulation in both HeLa and BMDC cells (Figure 5I,J). These results indicate that PS activates the cGAS‐IFN‐I signaling pathway.

### 2.5 PS Suppresses Tumor Growth by Inducing cGAS‐Dependent Anti‐Tumor Immunity

**Figure 6:**
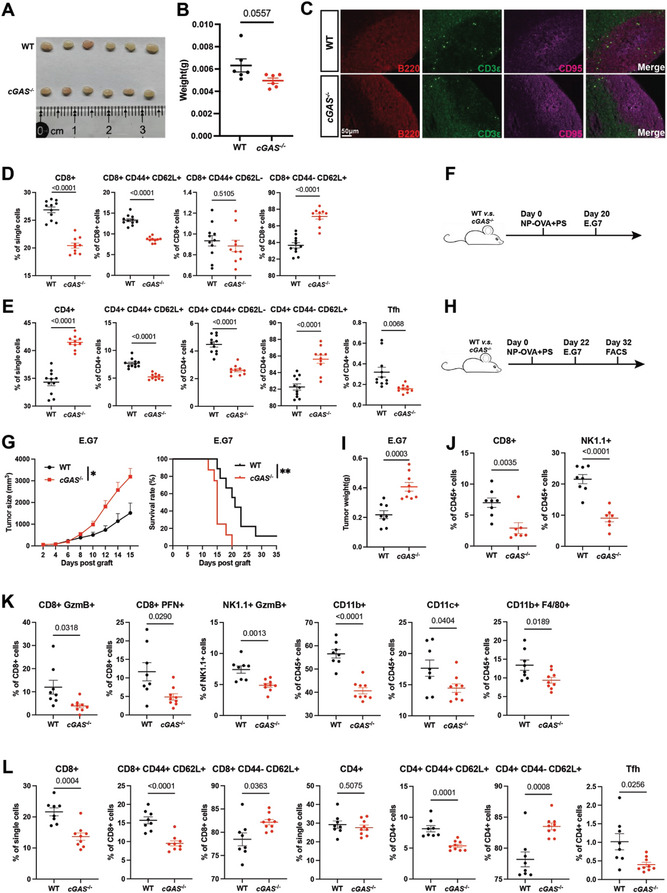
PS suppresses tumor growth by inducing cGAS‐dependent anti‐tumor immunity. A–C) WT and cGAS^−/−^ mice were immunized on day 0 with 50 µg NP‐OVA+100 µg PS. (A,B) On day 9, subcutaneous lymph nodes were removed and organ weights were recorded (n = 6). (C) Immunofluorescence analysis of subcutaneous lymph node sections on day 9. Antibodies detecting B220, CD3ε, and CD95 were used (n = 6). Scale bars: 50 µm. D,E) WT and cGAS^−/−^ mice were immunized on day 0 with 50 µg OVA+100 µg PS. Percentage of CD8+, CD4+ T cells and their subsets in subcutaneous lymph nodes were assessed 6 days after immunization (n = 11+10). (F,G) WT and cGAS^−/−^ mice were immunized on day 0 with 50 µg NP‐OVA+100 µg PS. 20 days post immunization, mice were challenged s.c. with 3 × 10^6^ of E.G7 cells. Tumor growth and mice survival were monitored over time (n = 9+8). H–L) WT and cGAS^−/−^ mice were immunized on day 0 with 50 µg NP‐OVA+100 µg PS. 22 days post immunization, mice were challenged s.c. with 3 × 10^6^ of E.G7 cells. On day 32, tumors and draining lymph nodes were removed for analysis. (I) Weight of tumors (n = 8+9). (J) Percentages of CD8+ and NK1.1+ TILs in tumors (n = 8+7). (K) Percentages of CD8+ and NK1.1+ TILs expressing GzmB or PFN, and CD11b+, CD11c+ cell types in tumors (n = 8+9). (L) Percentages of CD8+, CD4+ T cells and their subsets in draining lymph nodes (n = 8+9). Data are presented as mean ± SEM. *P* values were calculated by Student's t test (B,D,E,I,J,K,L), two‐way ANOVA (G tumor growth) and log‐rank test (G survival). ^*^ *p* < 0.05, ^**^ *p* < 0.01.

To determine if PS functions through cGAS in vivo, we analyzed the lymph nodes of cGAS knockout (cGAS^−/−^) and wild‐type mice following PS immunization. Our findings showed no notable differences in lymph node size and weight (**Figure** **6**A,B), germinal center formation (Figure 6C), or antibody production (Figure S7A, Supporting Information). However, examination of immune cell subsets in the lymph nodes revealed a significant reduction in CD8+ T cells and an increase in CD4+ T cells. Further analysis indicated a decrease in CD8+ and CD4+ T_CM_‐like cells, an increase in naive cells, and a significant reduction in Tfh cells (Figure 6D,E), indicating that the T cell response elicited by PS is cGAS‐dependent, while humoral immunity remains unaffected upon cGAS deletion.

When tumors were inoculated into unimmunized cGAS^−/−^ and wild‐type mice, no discernible differences in tumor growth were observed (Figure S7B, Supporting Information). However, in NP‐OVA/PS immunized mice (Figure 6F), cGAS^−/−^ mice exhibited significantly higher tumor volume and weight compared to the control group, accompanied by a significant reduction in survival rate (Figure 6G,I). Analysis of immune cell infiltration in tumors showed a significant decrease in CD8+ and NK1.1+ TILs in cGAS^−/−^ mice (Figure 6J). Additionally, CD8+ and NK1.1+ TILs from cGAS^−/−^ mice expressed lower levels of GzmB or PFN, and there was a reduction in CD11b+ and CD11c+ cells (Figure 6K). In tumor‐draining lymph nodes, there was a decrease in CD8+ T cells in cGAS^−/−^ mice, along with reduced CD8+ and CD4+ T_CM_‐like cells, increased naive cells, and a significant decrease in Tfh cells (Figure 6L). These findings suggest that activation of the cGAS‐IFN‐I pathway by PS enhances the T cell response, particularly by increasing CD8+ T_CM_‐like cells, leading to an enhanced anti‐tumor response.

### 2.6 PS Triggers cGAS Activation Via Cell Death

**Figure 7:**
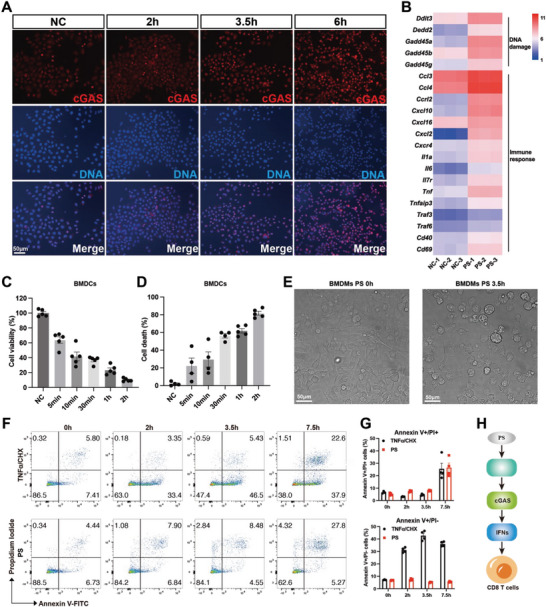
PS triggers cGAS activation via cell death. A) Immunofluorescence analysis of HeLa cells post PS stimulation. The antibody detecting cGAS and Hoechst detecting DNA were used. Scale bars: 50 µm. B) Heatmap showing the feature genes related to DNA damage and immune response in BMDCs post PS stimulation by RNA‐Seq analysis (n = 3). C) ATP‐based cell viability of BMDCs post PS stimulation (n = 5). D) Lactate dehydrogenase (LDH)‐release‐based cell death of BMDCs post PS stimulation (n = 4 or 5). E) Morphology of BMDMs upon PS stimulation. Scale bars: 50 µm. F,G) Flow‐cytometry analysis of annexin V and propidium iodide staining of BMDCs post TNFα plus cycloheximide (TNFα/CHX) or PS stimulation (n = 4). H) Proposed model of PS function in T cell response. Data are presented as mean ± SEM.

Recent studies have shown that engulfed foreign DNA from dying cells or damaged tissues, as well as self‐DNA leaking from mitochondria or the nucleus, can act as an intracellular danger‐associated molecular pattern (DAMP) that triggers the innate immune response.^[^ ^31^, ^32^ ^]^ Both in vitro and in vivo evidence robustly supports that cGAS is a key cytoplasmic DNA sensor across various cell types.^[^ ^33^, ^34^, ^35^ ^]^ Consequently, we investigated the localization of cGAS and DNA in PS‐treated HeLa cells. Immunofluorescent staining showed patternal changes in DNA contents following PS treatment, with a pronounced association between DNA and cGAS (**Figure** **7**A). Moreover, the temperature‐dependent cellular thermal shift assay demonstrated that PS does not alter the thermal stability of STING and cGAS proteins in HeLa cell lysate, indicating no direct interaction between PS and these proteins (Figure S8A, Supporting Information). Collectively, these findings indicate that PS activates cGAS through accesing self‐derived DNA.

To elucidate the underlying mechanism of how PS activates the cGAS pathway, we explored pathways that might influence DNA exposure. In addition to IFN‐I signaling, RNA‐Seq results indicated an upregulation of DNA damage and immune response genes following PS stimulation (Figure 7B). ATP‐based cell viability assays and lactate dehydrogenase (LDH) release assays in BMDCs confirmed cell death post‐PS treatment (Figure 7C,D). Consistently, we observed the characteristic morphology of dying cells with membrane rupture in BMDMs (Figure 7E; Video S1, Supporting Information) and HeLa cells (Video S2, Supporting Information).

We examined the mode of PS‐induced cell death, focusing on apoptosis, necroptosis, and pyroptosis, which are among the most well‐understood forms of cell death. Flow cytometry analysis of annexin V and propidium iodide staining confirmed that PS‐induced cell death was distinct from TNFα/cycloheximide (CHX)‐induced apoptosis (Figure 7F,G). TNFα plus SMAC mimetic and the caspase inhibitor zVAD (TSZ) induce necroptosis by the RIPK1‐RIPK3‐MLKL axis.^[^ ^36^ ^]^ We measured p‐MLKL levels in various cell types after PS stimulation. Interestingly, p‐MLKL was absent in PS‐treated cells, while treatment with TSZ significantly increased p‐MLKL levels (Figure S8B–D, Supporting Information). This suggests that PS‐induced cell death was unlikely through necroptosis. Gasdermins are a family of pore‐forming effector proteins that cause membrane permeabilization and pyroptosis^[^ ^37^ ^]^ The RNA‐Seq results showed that GSDMD was highly expressed in BMDCs (Figure S8E, Supporting Information), and we next determined whether GSDMD was cleaved upon PS treatment. However, no significant differences were observed in the cleavage of GSDMD following PS stimulation (Figure S8F, Supporting Information). Moreover, the expression of IFN‐β and IL‐6 was unaffected in GSDMD‐deficient BMDCs (Figure S8G, Supporting Information). Additionally, the antibody production (Figure S8H, Supporting Information) and anti‐tumor response (Figure S8I,J, Supporting Information) of GSDMD‐deficient mice were similar to those of wild‐type mice. These findings suggest that PS‐induced cell death may not proceed via apoptosis or rely on p‐MLKL or GSDMD, and it could be experiencing an unprogrammed progression.

## 3 Discussion

In this study, we conducted an extensive screening of 6800 compounds and identified PS as a potent adjuvant. PS was found to stimulate robust humoral and cellular immune responses, enhancing antibody production and T cell activity. Mechanistically, PS activated the cGAS‐IFN‐I pathway, leading to an increase in CD8+ T_CM_‐like cells and improved CD8+ T cell‐mediated anti‐tumor immunity (Figure 7H). These findings underscore the vital role of PS in the development of vaccine adjuvants and cancer prevention.

PS is a bis‐β‐carboline alkaloid isolated from *Picrasma quassioides*. β‐carboline alkaloids have demonstrated various pharmacological benefits, including anti‐cancer, anti‐inflammatory, and antiviral effects.^[^ ^38^ ^]^ The development of total syntheses for PS and its derivatives^[^ ^39^ ^]^ enhances its availability and potential as an adjuvant. A comparison of these derivatives showed that PS had the most potent activity in inducing IFN‐β in BMDCs (Figure S2B, Supporting Information). These findings support further clinical research into the benefits of β‐carboline alkaloids.

IFN‐I plays a pivotal role in linking innate and adaptive immunity by promoting T_H_1 responses, particularly CTL activation, and the survival of memory T T cells.^[^ ^40^, ^41^, ^42^ ^]^ Our adjuvant discovery strategy has demonstrated that activating this pathway can be an effective selection method for vaccine adjuvant development. This approach offers the potential to investigate additional signals that shape adaptive immune responses. By stimulating the cGAS‐IFN‐I pathway, PS significantly boosts both humoral and cellular immune responses. These findings align with ongoing efforts to develop cGAS‐STING agonists as adjuvants.^[^ ^30^, ^34^, ^43^, ^44^ ^]^ Crucially, our studies show that PS immunization leads to the differentiation of tumor antigen‐specific CD8+ T_CM_‐like cells, in agreement with recent research indicating that the cGAS‐STING cascade fosters stem cell‐like CD8+ T cells and is vital for anti‐tumor immune responses^[^ ^45^ ^]^ and various anti‐tumor therapies depend on activating the cGAS‐STING pathway.^[^ ^32^, ^46^, ^47^, ^48^ ^]^ Exploring whether PS can be applied in therapeutic vaccinations against tumors and other diseases, such as viral and bacterial infections, is critically important.

Admittedly, a deeper understanding of the upstream mechanisms activating cGAS is essential. We noted that PS induced cell death across various cell types. The co‐localization of DNA with cGAS following PS treatment implies that cGAS activation may be triggered by the release of self‐derived DNA. Investigating the direct targets of PS will illuminate the cell death process and the role of self‐derived DNA in activating cGAS.

The discovery of PS as a new modality of vaccine adjuvant has provided a timely option for vaccine design and can be seamlessly integrated into existing vaccine techniques. For instance, the recent clinical approval of mRNA‐based vaccines for COVID‐19 prevention has significantly fueled interest in developing vaccines for both prophylactic and therapeutic purposes in cancer.^[^ ^49^, ^50^, ^51^ ^]^ However, the practical application of mRNA vaccines has been constrained by instability, inefficient in vivo delivery, and innate immunogenicity.^[^ ^52^, ^53^ ^]^ Studies have suggested that optimal immune responses could be achieved through combination with adjuvants. STING agonists, for example, have been employed as immunomodulators in conjunction with mRNA and peptide vaccines.^[^ ^54^, ^55^ ^]^ Given its high potency in inducing cellular immunity, PS could be incorporated into many existing or developing vaccine strategies to enhance the CTL response desired in tumor or virus vaccination.

## 4 Experimental Section

### Animal Information

All experimental procedures in mice were performed in compliance with the protocol approved by the Institutional Animal Care and Use Committee (IACUC) of Tsinghua University (23‐ZWW1).

Animals were maintained on the 12 h light/dark cycle with chow diet and water available ad libitum. Mice utilized in the experiments were at the age of 6–12 weeks. C57BL/6 and CD‐1 mice were purchased from Charles River International. CD45.1 mice were purchased from Tsinghua University. cGAS^−/−^ mice were kindly gifted by Prof. Yonghui Zhang of Tsinghua University. IFN‐α/βR^−/−^ mice were kindly gifted by Prof. Gong Cheng of Tsinghua University and have been backcrossed onto the C57BL/6 background for more than six generations. GSDMD^−/−^ mice were kindly gifted by Prof. Wanli Liu of Tsinghua University.

### Reagents

NP‐OVA, NP‐KLH, and NP‐BSA were purchased from LGC Biosearch Technologies. OVA was purchased from Sigma‐Aldrich. Imject Alum was purchased from Thermo Fisher Scientific. PS was purchased from BioBioPha. 6800 compounds screened in this research and PS derivatives were kindly gifted by Prof. Xiaoguang Lei of Peking university. TNFα was purchased from Sinobiological. Cycloheximide was purchased from Sigma‐Aldrich. SMAC mimetic (SM‐164) was purchased from Beyotime. The pan‐caspase inhibitor zVAD (Z‐VAD‐FMK) was purchased from Meilunbio.

### Mouse Immunizations and Tumor Challenges

Mice were immunized with antigen (50 µg), such as NP‐OVA and DMSO or antigen in combination with different adjuvants suspended in PBS, with a final injection volume of 100 µL. The dose of Imject Alum, which contained aluminum hydroxide (40 mg mL^−1^) and magnesium hydroxide (40 mg mL^−1^), was 25 µL. The dose of PS dissolved in DMSO (40 mg mL^−1^) was 100 µg (2.5 µL), unless otherwise stated. The dose of DMXAA dissolved in DMSO (10 mg mL^−1^) was 55 µg (5.5 µL), which was used as equal molar amount as PS. When DMXAA was used as adjuvant, 100 µg PS (2.5 µL) was diluted in 5.5 µL DMSO. All injections were performed subcutaneously (s.c.) on double flanks.

Several days post‐immunization, mice were challenged s.c. on the left flank with viable E.G7, B16‐OVA, or MC38‐OVA cells, as indicated. Tumor sizes were measured every 2 days by electronic calipers and given as length (mm) × width (mm) × width (mm)/2. Mice with tumors larger than 20 mm on the longest axis were euthanized.

To deplete CD8+ or CD4+ T cells, 200 µg anti‐CD8 (Bio X cell; Cat# BE0061) or anti‐CD4 (Bio X cell; Cat# BE0119) antibody was given via intraperitoneal injection at the indicated time points, respectively. To block the IFN‐I signaling, 200 µg anti‐IFNAR‐1 (Bio X cell; Cat# BE0241) antibody was given via intraperitoneal injection on day −1 and day 1 after immunization, 400 µg anti‐IFNAR‐1 antibody was given on the day of immunization.

### Antibodies for Immunostaining

Primary antibodies used for immunostaining were rat anti‐CD45R (BD Biosciences, Cat# 557 390), FITC anti‐CD3ε (Biolegend; Cat# 100 306), PE/Cy7 anti‐CD95 (Biolegend; Cat# 152 618), cGAS (Cell Signaling Technology; Cat# 15 102). In addition, Alexa Fluor dye‐conjugated secondary antibodies were from Thermo Fisher Scientific. DNA was stained by Hoechst 33 258 (Solarbio; Cat# C0021) according to the manufacturer's instructions.

### Cell Culture and Stimulation

For the in vitro cultures of bone marrow‐derived dendritic cells (BMDCs), bone‐ marrow cells were collected from the mouse femurs. The cells were cultured in RPMI‐1640 (Sigma)/10% heat‐inactivated FBS (NEWZERUM) / 100 U mL^−1^ penicillin and streptomycin (Gibco)/20 ng mL^−1^ GM‐CSF (SinoBiological)/10 ng mL^−1^ IL‐4 (SinoBiological) as previously described.^[^ ^56^, ^57^ ^]^ Non‐adherent BMDCs were harvested and used for experiments on day 7 of differentiation.

For the in vitro cultures of bone marrow‐derived macrophages (BMDMs), bone marrow cells were harvested from the mouse femurs. The cells were cultured in DMEM (Corning)/10% heat‐inactivated FBS/100 U mL^−1^ penicillin and streptomycin/10 ng mL^−1^ M‐CSF (PeproTech) for 5 days before the experiments.

HeLa/HEK293T/B16‐OVA/Bv‐2/MC38‐OVA cells were cultured in DMEM/10% heat‐inactivated FBS/100 U ml^−1^ penicillin and streptomycin. E.G7 and Thp1 cells were cultured in RPMI‐1640/10% heat‐inactivated FBS/100 U mL^−1^ penicillin and streptomycin. B16‐OVA, E.G7, and MC38‐OVA were generously provided by Prof. Zhongjun Dong, Tsinghua University. All cells were cultured at 37 °C and 5% CO_2_. Cells were treated with PS (10 µm) for 8 h, unless otherwise stated.

### ELISA Analysis

To measure antibody titers, serum samples were collected from whole blood by centrifugation. ELISA plates were coated with 5 µg mL^−1^ NP‐BSA in PBS at 4 °C, overnight. After washing, plates were blocked with 0.3% gelatin in PBS for 1 h and 1% BSA in PBS for 1 h, followed by addition of diluted serum into each well and incubated for 2 h at room temperature. Plates were then washed and incubated with HRP conjugated goat anti‐mouse IgM (Boster), IgG, IgG1, IgG2b, IgG2c, or IgG3 (Thermo Fisher Scientific) antibodies for 1 h at 37 °C. Then 1 × TMB substrate solution (Thermo Fisher Scientific) was added (100 mL well^−1^) into each well and incubated for 15 min at room temperature, followed by the addition of 1 m H_3_PO_4_ (50 µL well^−1^) to terminate the reaction. The plates were read at 450 and 570 nm using the ELISA plate reader (Bio‐Rad). Readings at 570 nm were subtracted from the readings at 450 nm.

For measurement of IFN‐β of BMDCs, cell culture supernatant was centrifuged to remove debris and stored at −80 °C. The samples were then measured by the IFN‐β ELISA kit (BioLegend; Cat# 439 407) according to the manufacturer's instructions.

### Western Blotting

Cells were lysed by RIPA buffer containing protease inhibitor cocktail (TargetMol). The samples were subjected to sodium dodecyl‐sulfate (SDS)‐polyacrylamide gel electrophoresis (PAGE) and blotted using the indicated antibodies. Primary antibodies include: rabbit anti‐GAPDH (Cell Signaling Technology; Cat# 5174), rabbit anti‐TBK1 (Cell Signaling Technology; Cat# 3504), rabbit anti‐p‐TBK1 (Cell Signaling Technology; Cat# 5483), rabbit anti‐HSP90 (Cell Signaling Technology; Cat# 4874), rabbit anti‐cGAS (Cell Signaling Technology; Cat#15 102), rabbit anti‐STING (Cell Signaling Technology; Cat#13 647) and rabbit anti‐GSDMD (abcam; ab209845). The temperature‐dependent cellular thermal shift assay was performed as follows: HeLa lysate was equally devided, then 10 µm PS or DMSO were added and incubated at 4 °C for 30 min. The samples were subjected to a gradient temperature heating for 3 min followed by high speed centrifugation, and the supernatant was collected for SDS‐PAGE and western blot detection.

### Flow Cytometry

Single cells suspension from lymph nodes were blocked with anti‐CD16/32 antibody (Biolegend, Cat#101 302), then stained with cocktails of following fluorescently conjugated antibodies (BioLegend): CD19‐APC‐Cy7, CD4‐V450, CD8‐FITC, CD44‐AF700, CD62L‐APC, CXCR5‐PE, PD‐1‐PE‐Cy7. Single cells suspension from tumors or draining inguinal lymph nodes were stained with Fixable Viability Dye eFluor 780 (ThermoFisher Scientific, Cat#65‐0865‐14) to determine the viability of cells prior to antibodies staining. Cells were blocked then stained with cocktails of following fluorescently conjugated antibodies: CD45‐AF700 (Thermo Fisher Scientific), CD3‐PE‐Cy7 (BioLegend), CD4‐BV510 (BioLegend), CD8‐eFluor 450 (Thermo Fisher Scientific), NK1.1‐FITC (BioLegend), CD11b‐BV510 (BioLegend), F4/80‐ eFluor 450 (Thermo Fisher Scientific), CD11c‐FITC (BioLegend). For intracellular staining, cells were first stained with antibodies to cell‐surface markers for 30 min, then fixed and permeabilized with fixation and permeabilization buffer (ThermoFisher Scientific) and stained with GzmB‐APC (BioLegend) and perforin‐PE (BioLegend). For intracellular cytokine staining, cells were stimulated with 50 ng mL^−1^ PMA (Sigma) and 2 µg mL^−1^ ionomycin (Sigma) with Monensin (BioLegend) and Brefeldin A (BioLegend) for 4 h. Cells were then stained with IFN‐γ‐PE (BioLegend) and TNF‐α‐APC (BioLegend) after fixation and permeabilization. Samples were analyzed using LSR II (BD Biosciences) and FlowJo v10.8.1 software (BD Biosciences).

### Cell‐Death Assays

Cell death was assessed by measuring LDH release using a LDH cytotoxicity assay kit (Leagene; Cat# CT0027) following the manufacturer's protocol. Cell viability was assessed by measuring ATP levels using an ATPlite kit (PerkinElmer, Cat#6 016 941) following the manufacturer's protocol. The control cells were considered as 100%, and cell death was calculated by the decrease in the number of viable cells. Cell death due to apoptosis was assessed by Annexin V‐FITC/PI Apoptosis Detection kit (Vazyme, Cat#A211) following the manufacturer's protocol.

### Gene‐Expression Assays

RNA was extracted using TRIzol reagent and was subjected to reverse transcription to make cDNA. Gene expression was assayed by qPCR using 2 × M5 HiPer SYBR Premix Es Taq (Mei5bio) on Step One Plus Real‐Time PCR System (Applied Biosystems). The primers used for each gene examined are listed below.

mIFNB1: CACAGCCCTCTCCATCAACTA (forward) and CATTTCCGAATGTTCGTCCT (reverse);

hIFNB1: ATGACCAACAAGTGTCTCCTCC (forward) and GGAATCCAAGCAAGTTGTAGCTC (reverse);

mIL6: GCTACCAAACTGGATATAATCAGGA (forward) and CCAGGTAGCTATGGTACTCCAGAA (reverse);

hIL6: ACTCACCTCTTCAGAACGAATTG (forward) and

CCATCTTTGGAAGGTTCAGGTTG (reverse);

mcGAS: CAGGAAGGAACCGGACAAGC (forward) and

CCGACTCCCGTTTCTGCATT (reverse);

hcGAS: ACATGGCGGCTATCCTTCTCT (forward) and

GGGTTCTGGGTACATACGTGAAA (reverse);

mHPRT: TGAAGAGCTACTGTAATGATCAGTCAAC (forward) and

AGCAAGCTTGCAACCTTAACCA (reverse);

hCyclophilin: GGTCCCAAAGACAGCAGAAA (forward) and

GTCACCACCCTGACACATAAA (reverse).

### EC_50_ Determination of PS in Inducing Cytokines

EC_50_ (half‐maximal effective concentration) of PS was assessed in BMDCs with indicated dose for 8 h through qPCR. Curve was obtained using transformation, normalization and nonlinear regression by Graphpad Prism.

### Toxicity Assessment of PS‐Mediated Vaccination

C57BL/6 mice were vaccinated on day 0 with NP‐OVA (50 µg) in combination with DMSO (2.5 µL) or NP‐OVA (50 µg) in combination with PS (2.5 µL, 100 µg). Their mortality and body weight were monitored during the entire time course. On day 7, spleens, kidneys, lungs, livers and skin were analyzed by HE staining.

### RNA‐Seq and Data Analysis

The total RNA was extracted using TRIzol reagent and processed for RNA sequencing. The RNA sequencing data were mapped to the mouse reference genome by HISAT2/Bowtie2 tool. Differential expression genes upregulated or downregulated by 2‐fold or higher were analyzed further, with the adjusted *P* value (padj) less than 0.05. The raw sequence data has been deposited in the Sequence Read Archive (SRA) database with accession number PRJNA1041496.

### Pharmacokinetics Parameters of PS in Mice

The pharmacokinetics parameters of PS were tested by a single intravenous (i.v.) administration in 7‐week‐old male CD‐1 mice. Blood samples were collected at 0.083, 0.25, 1, 2, 4, 6, 8, and 24 h post administration through orbital venous plexus by capillary, EDTA‐K2 anticoagulant. The PS concentration in plasma was quantified by LC‐MS/MS, and data analysis was conducted using PKsolver2.0. T_1/2_, half‐life; Cmax, maximum plasma concentration; AUC, area under the curve.

### Statistics

Student's t test, ANOVA test or log‐rank test were performed using GraphPad Prism. Statistical details of the experiments are included in the figure legends. Graphical data was shown as mean values with error bars indicating the SEM. *P* values of < 0.05 (^*^), < 0.01 (^**^), < 0.001 (^***^), < 0.0001 (^****^) indicated significant differences between groups.

## Conflict of Interest

The authors declare no conflict of interest.

## Author Contributions

X.D., M.S., F.G., and X.Q. contributed equally to this work. W.Z., X.L., and X.D. conceived and designed the project. W.Z. and X.L. supervised and managed the project. X.D., M.S., F.G., X. Q., and W.L. performed and analyzed the experiments. Q.W. synthesized the chemicals. W.Z. and X.D. wrote the manuscript with the assistance of X.L., F.G., M.S., X.Q., and W.L.

## Supporting information

Supporting Information

Supplemental Video1

Supplemental Video2

## Data Availability

All data supporting the findings of the present study are available within the paper or from the corresponding author upon request.
