# Supplementary material for: Picrasidine S Induces cGAS‐Mediated Cellular Immune Response as a Novel Vaccine Adjuvant
Source: Adv Sci (Weinh). 2024 Jun 20;11(32):2310108. doi: 10.1002/advs.202310108 (PMC11348072; doi:10.1002/advs.202310108)
Supplement: Supplementary file 1 — Supporting Information [file ADVS-11-2310108-s003.docx]

Supplemental Figures


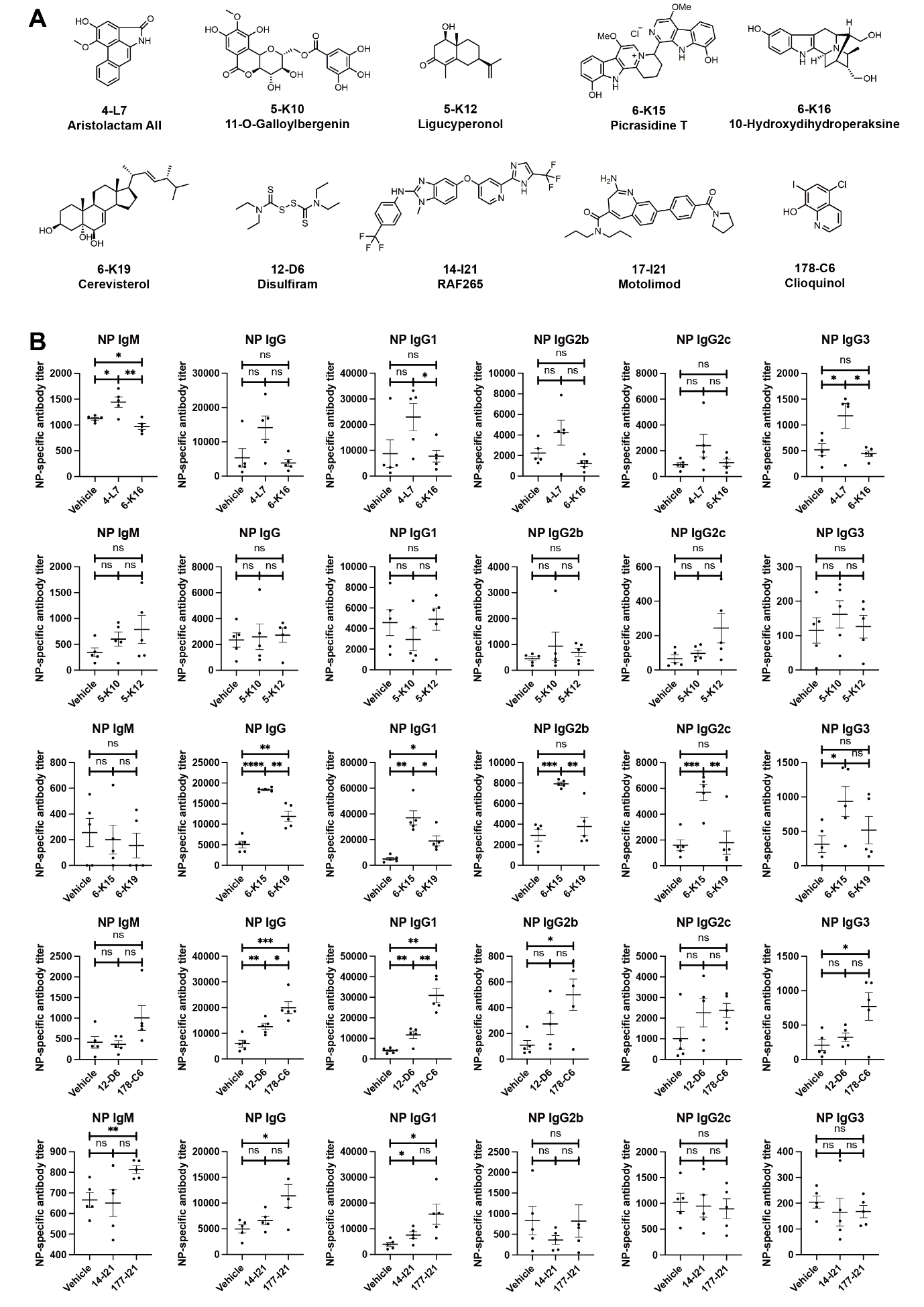


**Figure S1. PT enhances humoral immunity in vaccination**

**(A)** Chemical structure of drugs.

**(B)** C57BL/6 mice were immunized with 50 μg NP-KLH or 50 μg NP-KLH admixed with 125 μg indicated drugs on day 0. Serum NP-specific antibody titers in IgM, IgG, IgG1, IgG2b, IgG2C and IgG3 isotypes on day 14 were examined by ELISA (n=5).

Data are presented as mean ± SEM. *P* values were calculated by Student’s t test **(B)**. ns (nonsignificant) P > 0.05, *P < 0.05, **P < 0.01, ***P < 0.001, ****P < 0.0001.

**
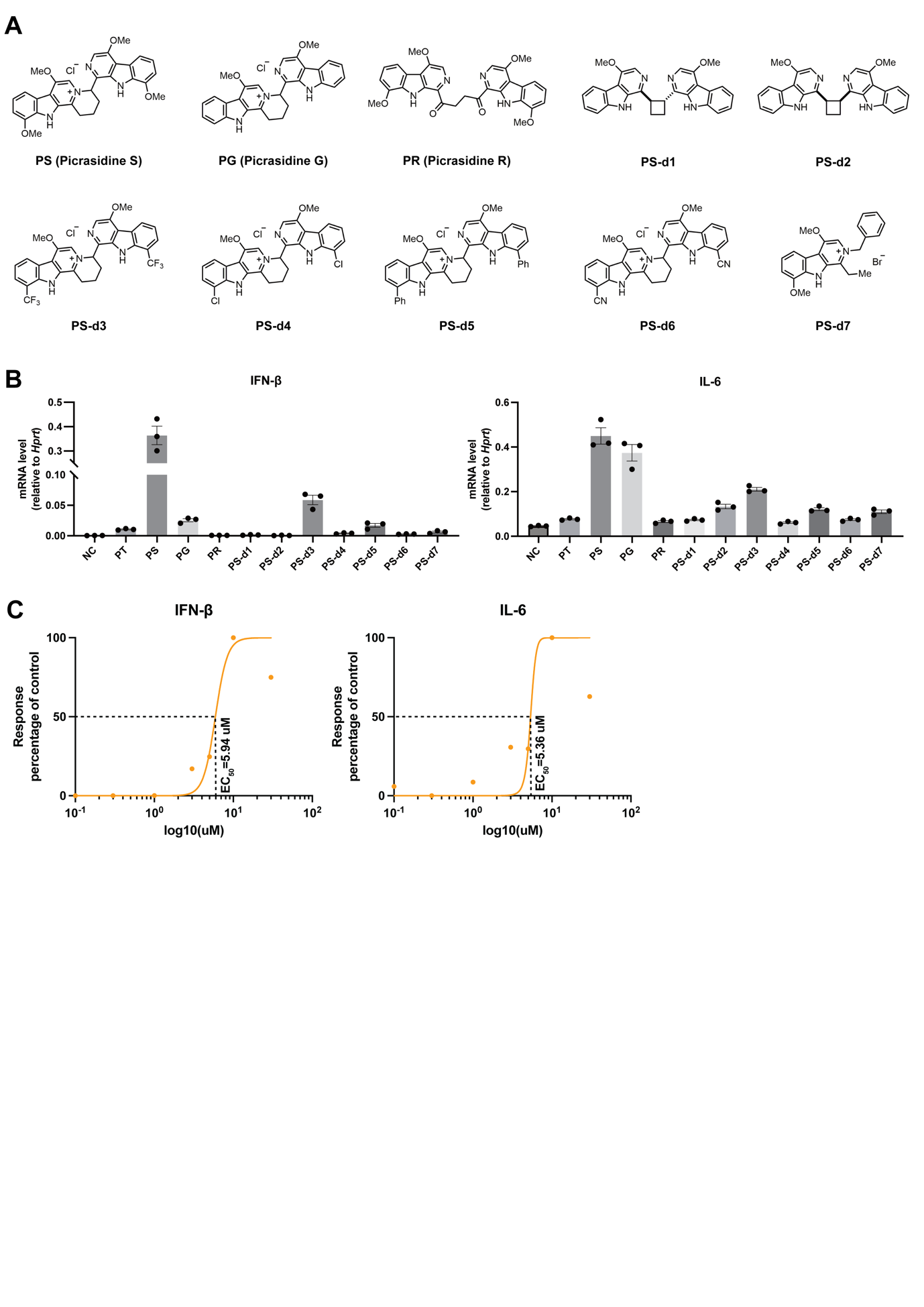
Figure S2. PS shows the highest activity in IFN-β induction**

**(A)** Chemical structure of PS and its derivatives.

**(B)** Expression levels of IFN-β and IL-6 in BMDCs post drug stimulation were determined by the qPCR analysis (n=3).

Data are presented as mean ± SEM.

**(C)** EC_50_ of PS in BMDCs was determined by the qPCR analysis with indicated PS concentrations for 8 h (n=3).


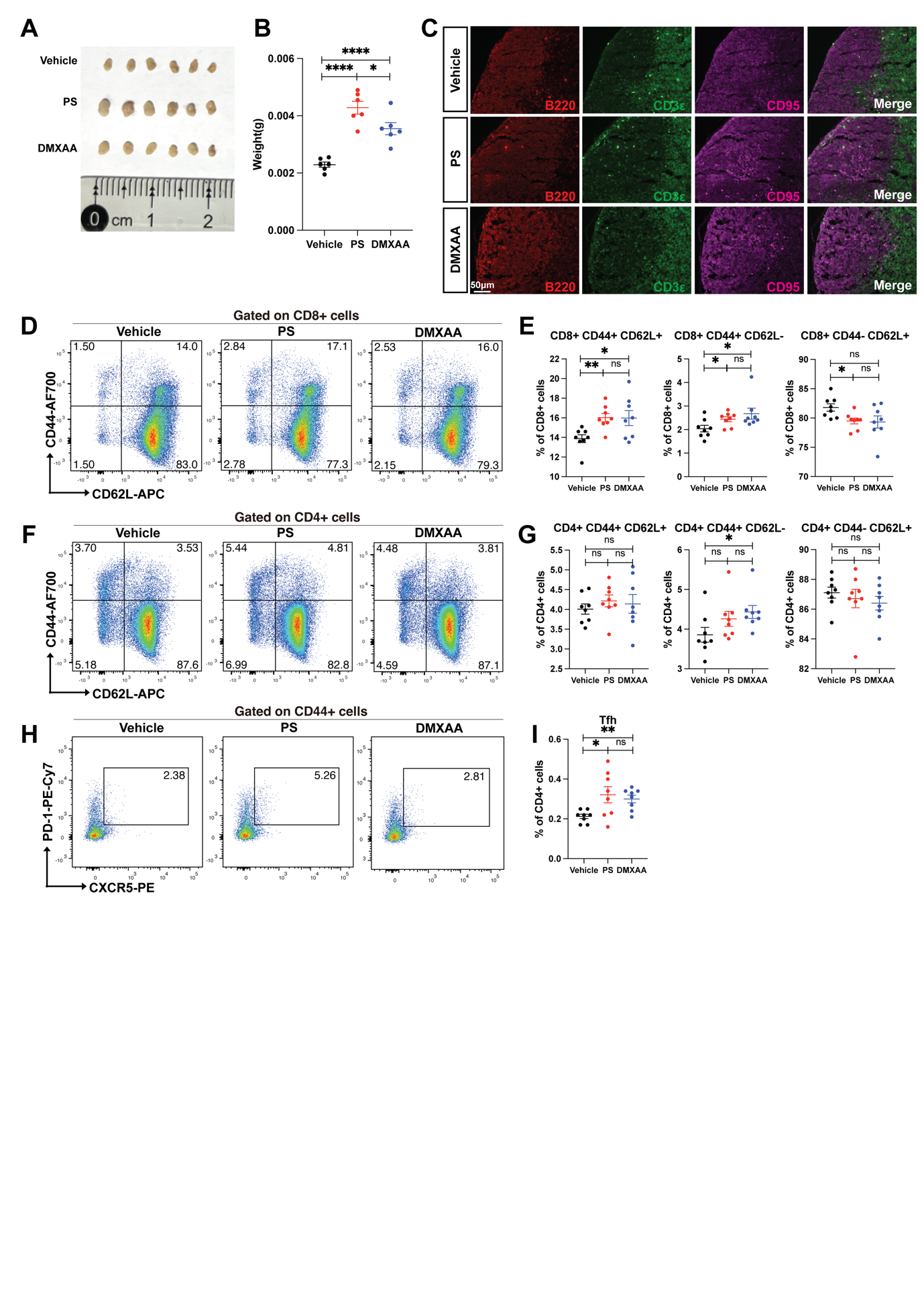


**Figure S3. PS enhances cellular immunity in vaccination, related to Figure 3**

**(A-C)** C57BL/6 mice were immunized on day 0 with 50 μg OVA, 50 μg OVA+100 μg PS or 50 μg OVA+55.4 ug DMXAA (282.29g/mol). **(A and B)** On day 9, subcutaneous lymph nodes were removed and organ weights were recorded (n=6). **(C)** Immunofluorescence analysis of subcutaneous lymph node sections on day 9. Antibodies detecting B220, CD3ε and CD95 (which marks germinal center B cells) were used (n=6). Scale bars: 50 μm.

**(D-I)** C57BL/6 mice were immunized on day 0 with 50 μg NP-OVA, 50 μg NP-OVA+100 μg PS or 50 μg NP-OVA+55.4 ug DMXAA (282.29g/mol). On day 9, subcutaneous lymph nodes were removed for flow-cytometry analysis (n=8). **(D-G)** The percentage of standard subsets of CD8+ and CD4+ T cells, including central memory (T_CM_, CD44+ CD62L+), effector memory (T_EM_, CD44+ CD62L-) and naïve (CD44- CD62L+). **(H and I)** The percentage of T follicular helper cells (Tfh, CD44+ CXCR5+ PD-1+).

Data are presented as mean ± SEM. *P* values were calculated by Student’s t test **(B, E, G, I)**. ns (nonsignificant) *P* > 0.05, **P* < 0.05, ***P* < 0.01, ****P* < 0.001, *****P* < 0.0001.


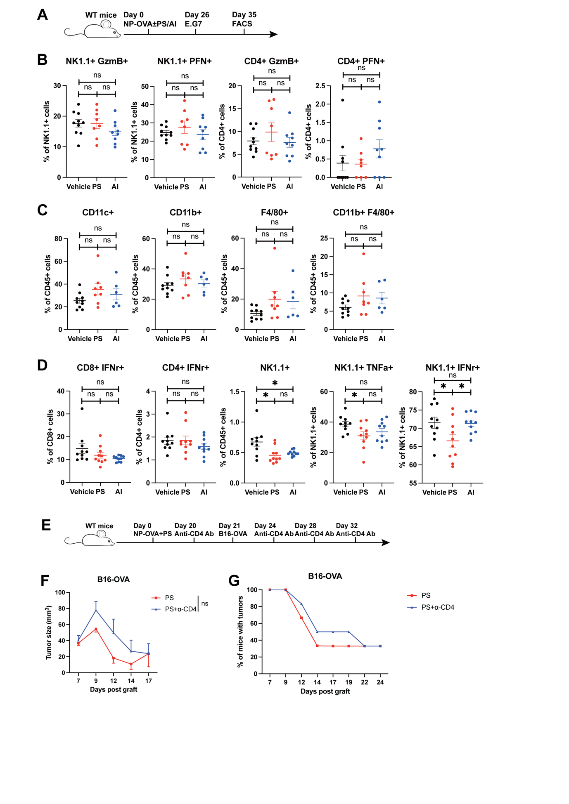


**Figure S4. PS promotes anti-tumor immunity in a CD8+ T cell-dependent manner, ﻿related to Figure 4**

**(A-D)** C57BL/6 mice were immunized on day 0 with 50 μg NP-OVA, 50 μg NP-OVA+100μg PS or 50 μg NP-OVA+25 μl alum. 26 days post immunization, mice were challenged s.c. with 3 × 10^6^ of E.G7 cells. On day 35, tumors and draining lymph nodes were removed for flow-cytometry analysis. **(B)** Percentages of NK1.1+, CD4+ TILs expressing GzmB or PFN in tumors (n=10+8+9). **(C)** Percentages of CD11c+, CD11b+ and F4/80+ cells in tumors (n=10+8+6). **(D)** Percentages of CD8+, CD4+, and NK1.1+ cells producing IFNr or TNFα induced by PMA and ionomycin in draining lymph nodes (n=10).

**(E-G)** C57BL/6 mice were immunized on day 0 with 50 μg NP-OVA+100 μg PS. 21 days post immunization, mice were challenged s.c. with 3 × 10^6^ of B16-OVA cells. CD4-depleting antibody was injected at the indicated time points. Tumor growth **(F)** and tumor incidence **(G)** were monitored over time (n=6).

Data are presented as mean ± SEM. *P* values were calculated by Student’s t test **(B-D)** and two-way ANOVA **(F)**. ns (nonsignificant) *P* > 0.05, **P* < 0.05.


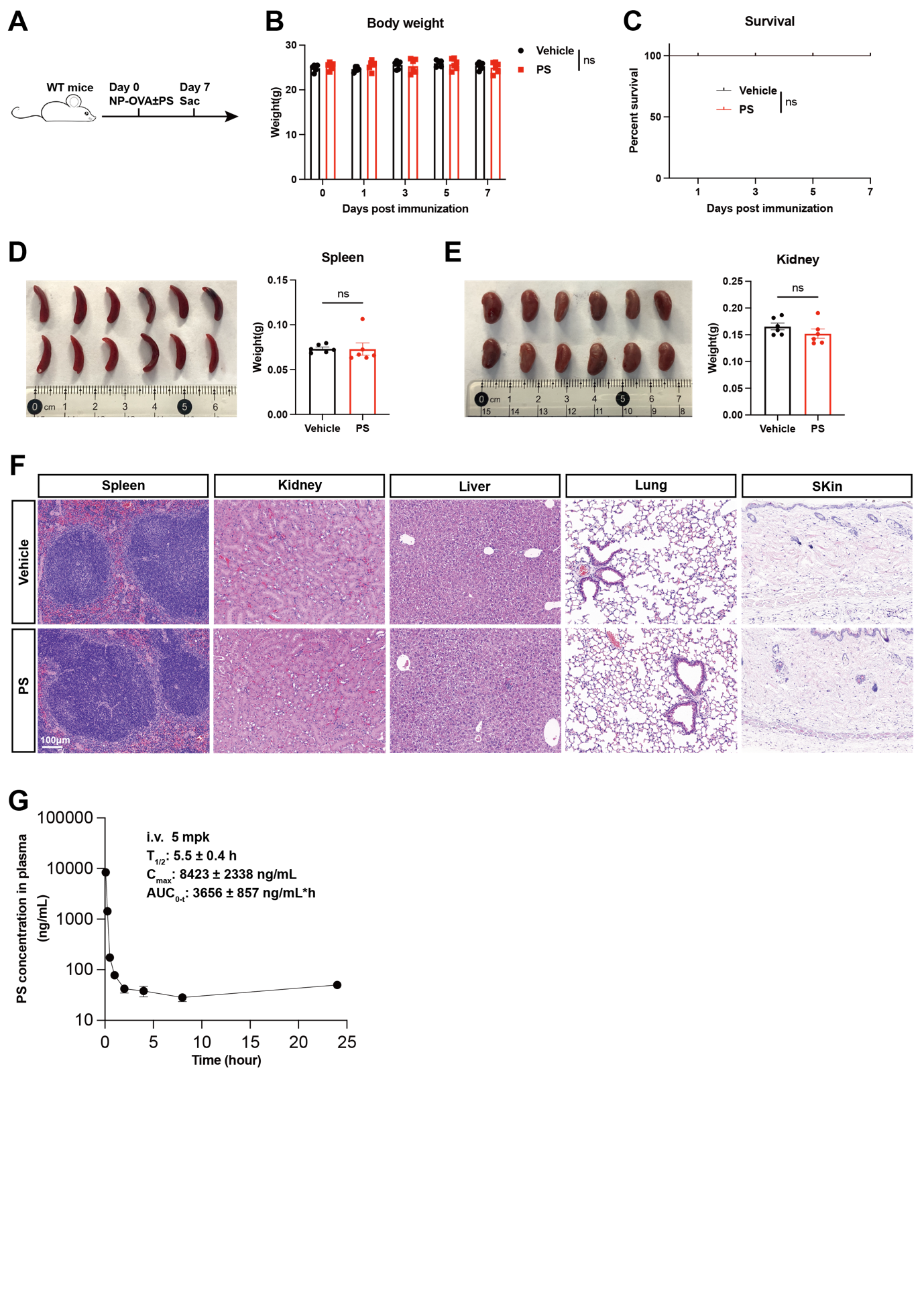


**Figure S5. ﻿Toxicity and pharmacokinetic evaluation of PS-mediated vaccination**

**(A-F)** C57BL/6 mice were vaccinated on day 0 with 50 μg OVA or 50 μg OVA+100μg PS (n=6). Their Body weights **(B)** and ﻿mortality **(C)** was monitored every two days. **(D-F)** On day 7, organs were removed to record weights **(D and E)** and for H&E staining **(F)**.

**(G)** Pharmacokinetics of PS in mice. T_1/2_, half-life; C_max_, maximum plasma concentration; AUC, area under the curve.

Data are presented as mean ± SEM. *P* values were calculated by Student’s t test **(B, D, E)** and log-rank test **(C)**. ns (nonsignificant) P > 0.05.


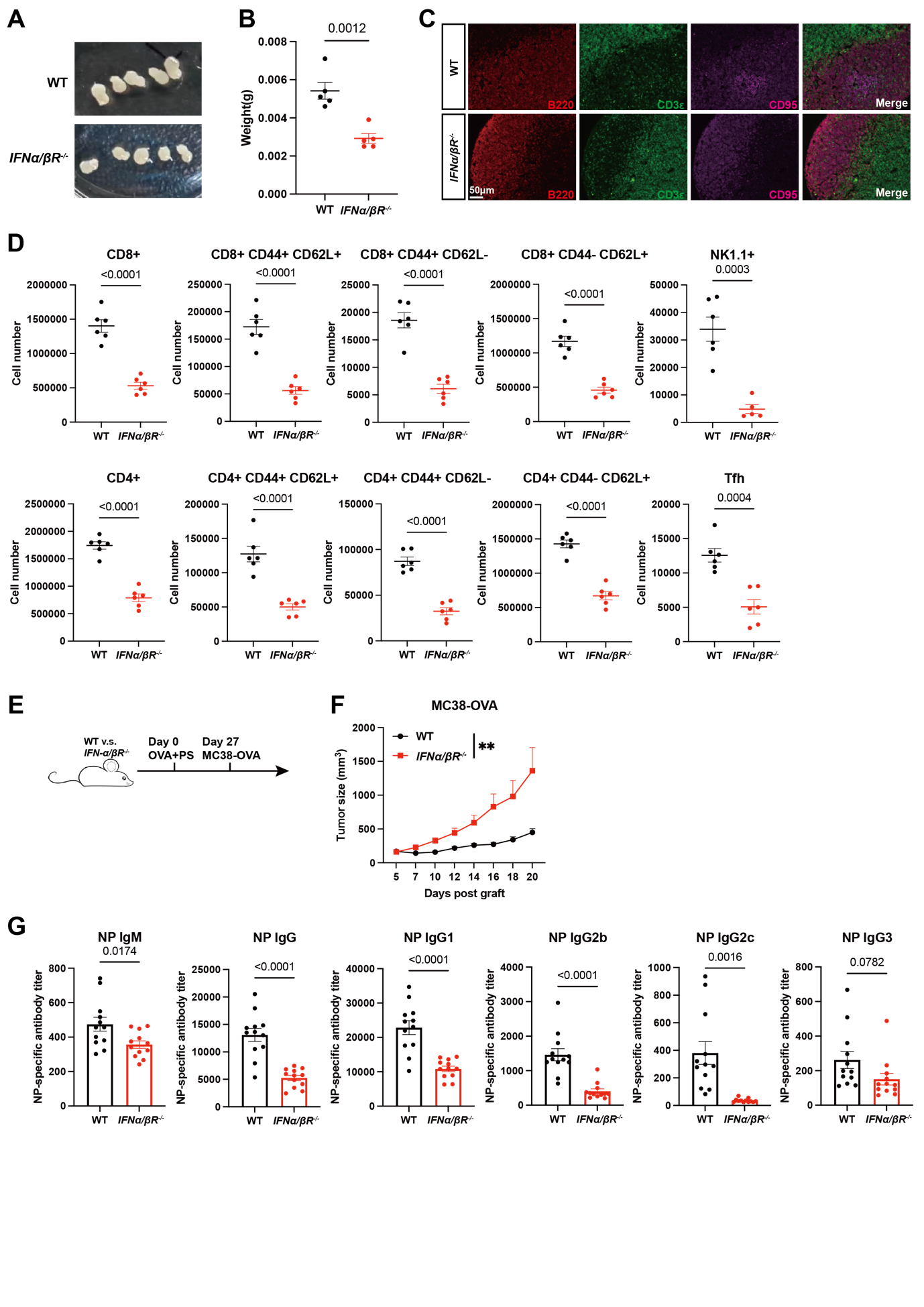


**Figure S6. IFN-I is essential to PS-induced cellular and humoral immunity**

**(A-D)** WT and IFNα/βR^-/-^ mice were immunized on day 0 with 50 μg OVA+100μg PS. On day 6, subcutaneous lymph nodes were removed for analysis. **(A and B)** Organ weights (n=5). **(C)** Immunofluorescence analysis of lymph node sections. Antibodies detecting B220, CD3ε and CD95 were used (n=6). Scale bars: 50 μm. **(D)** Cell number of CD8+, CD4+, NK1.1+ cells and their subsets in lymph nodes (n=6).

**(E and F)** WT and IFNα/βR^-/-^ mice were immunized on day 0 with 50 μg OVA+100μg PS. 27 days post immunization, mice were challenged s.c. with 2 × 10^6^ of MC38-OVA cells. Tumor growth were monitored over time (n=12+10).

**(G)** WT and IFNα/βR^-/-^ mice were immunized on day 0 with 50 μg NP-OVA+100μg PS. Serum NP-specific antibody titers in IgM, IgG, IgG1, IgG2b, IgG2C and IgG3 isotypes on day 14 were examined by ELISA (n=12).

Data are presented as mean ± SEM. *P* values were calculated by Student’s t test **(B, D, G)** and two-way ANOVA **(F)**. **P < 0.01.


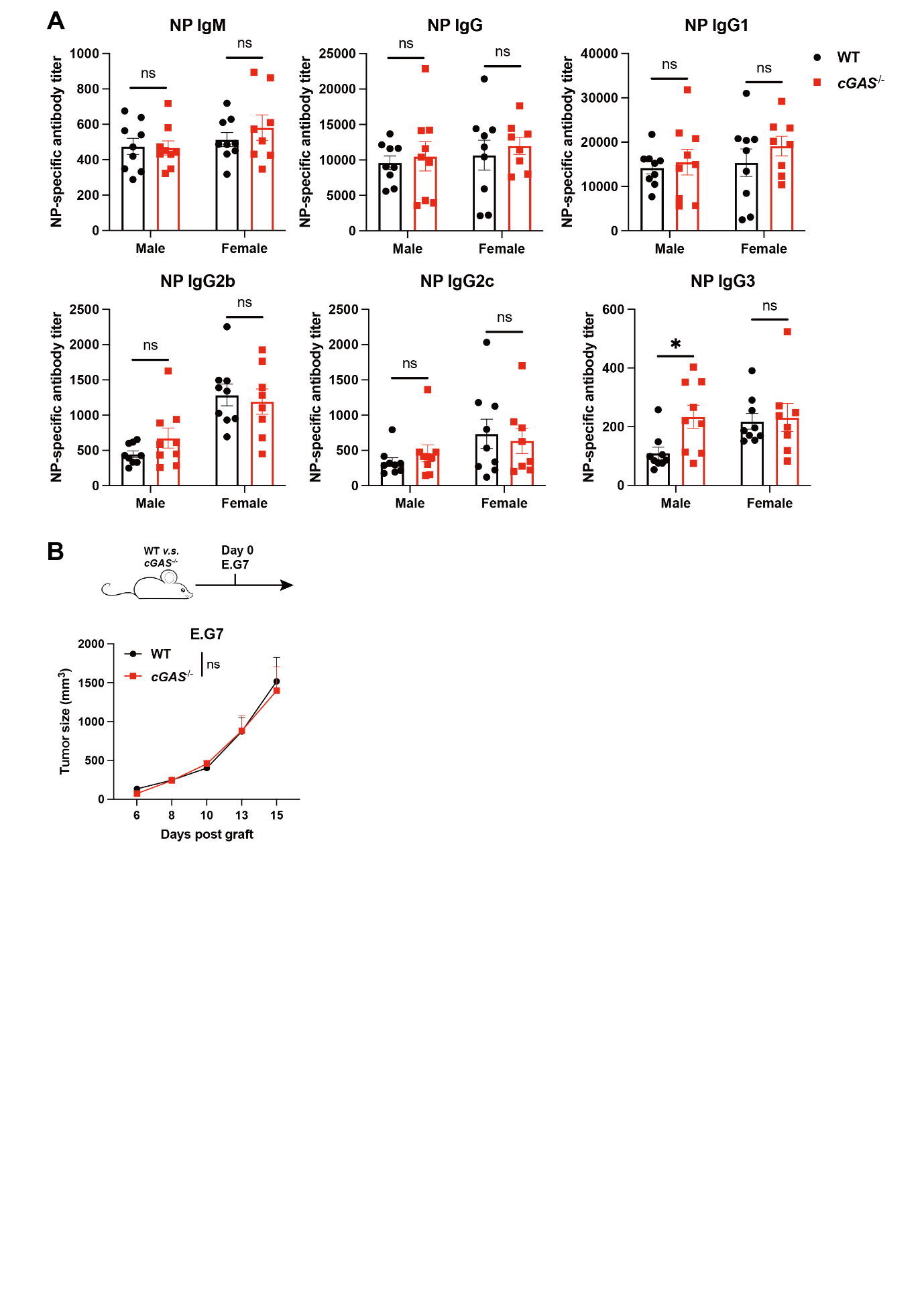


**Figure S7. cGAS is not essential to PS-induced humoral immunity**

**(A)** WT and cGAS^-/-^ mice were immunized on day 0 with 50 μg NP-OVA+100μg PS. Serum NP-specific antibody titers in IgM, IgG, IgG1, IgG2b, IgG2C and IgG3 isotypes on day 14 were examined by ELISA (n=9 or 8).

**(B)** WT and cGAS^-/-^ mice were challenged s.c. with 5 × 10^5^ of E.G7 cells. Tumor growth were monitored over time (n=6).

Data are presented as mean ± SEM. *P* values were calculated by Student’s t test **(A)** and two-way ANOVA **(B)**. ns (nonsignificant) P > 0.05, *P < 0.05.


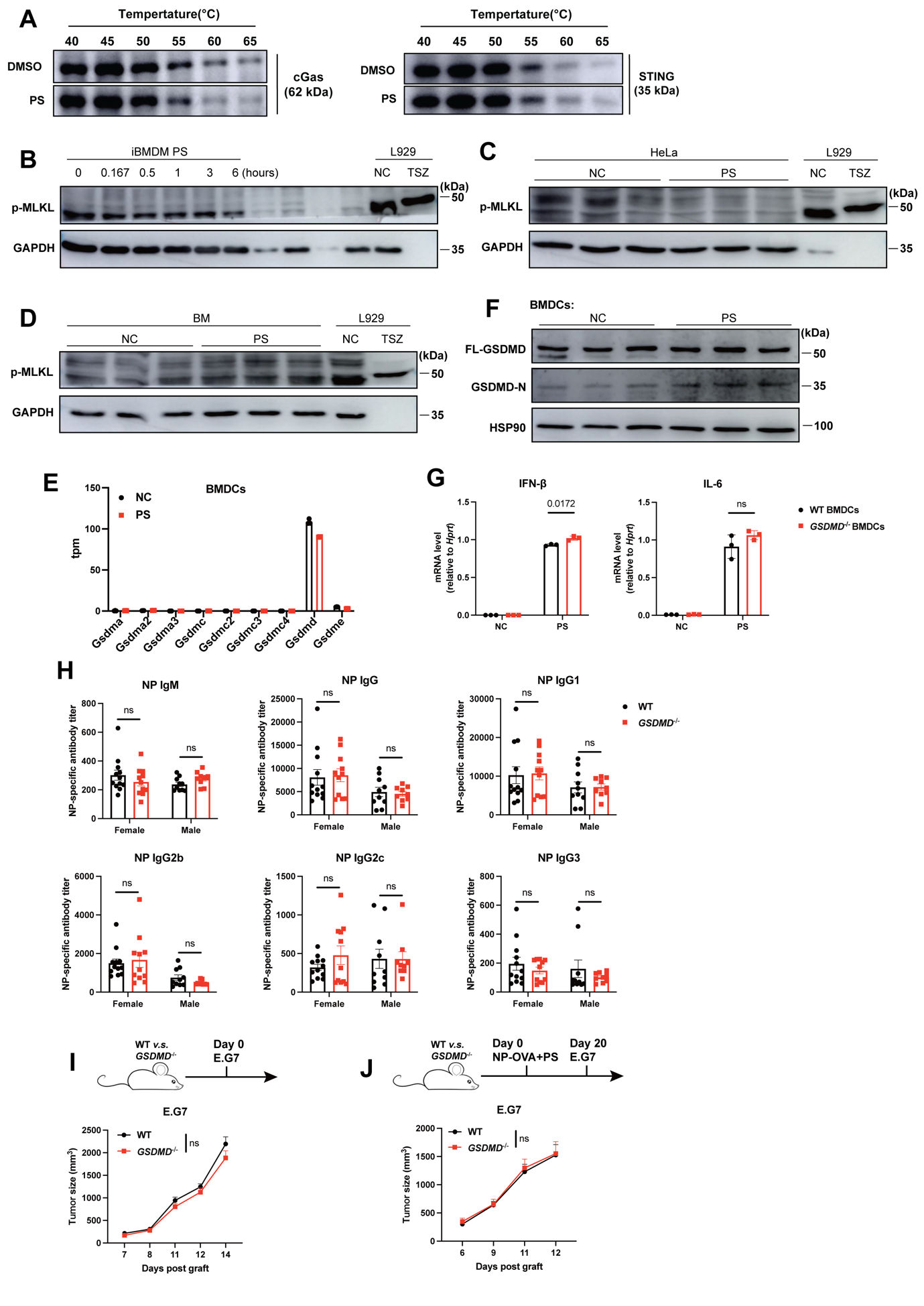


**Figure S8. PS-induced immunity is independent of p-MLKL and GSDMD**

**(A)** Immunoblot analysis of temperature-dependent cellular thermal shift assay in HeLa cells.

**(B-D)** Immunoblot analysis of iBMDM **(A)**, HeLa **(B)** and bone marrow (BM) cells **(C)** post PS stimulation. TNFα plus SMAC mimetic and the caspase inhibitor zVAD (TSZ)-induced L929 as a positive control.

**(E)** Expression levels of Gasdermins in BMDCs post PS stimulation by RNA-Seq analysis (n=3).

**(F)** Immunoblot analysis of BMDCs post PS stimulation.

**(G)** Expression levels of IFN-β and IL-6 in WT and GSDMD-deficient (GSDMD^-/-^) BMDCs post PS stimulation were determined by the qPCR analysis (n=3).

**(H)** WT and GSDMD^-/-^ mice were immunized on day 0 with 50 μg NP-OVA+100μg PS. Serum NP-specific antibody titers in IgM, IgG, IgG1, IgG2b, IgG2C and IgG3 isotypes on day 14 were examined by ELISA (Female: n=12+11; Male: n=10+9).

**(I)** WT and GSDMD^-/-^ mice were challenged s.c. with 1 × 10^6^ of E.G7 cells. Tumor growth were monitored over time (n=11+9).

**(J)** WT and GSDMD^-/-^ mice were immunized on day 0 with 50 μg NP-OVA+100μg PS. 20 days post immunization, mice were challenged s.c. with 3 × 10^6^ of E.G7 cells. Tumor growth were monitored over time (n=10+8).

Data are presented as mean ± SEM. *P* values were calculated by Student’s t test **(G and H)** and two-way ANOVA **(I and J)**. ns (nonsignificant) P > 0.05.


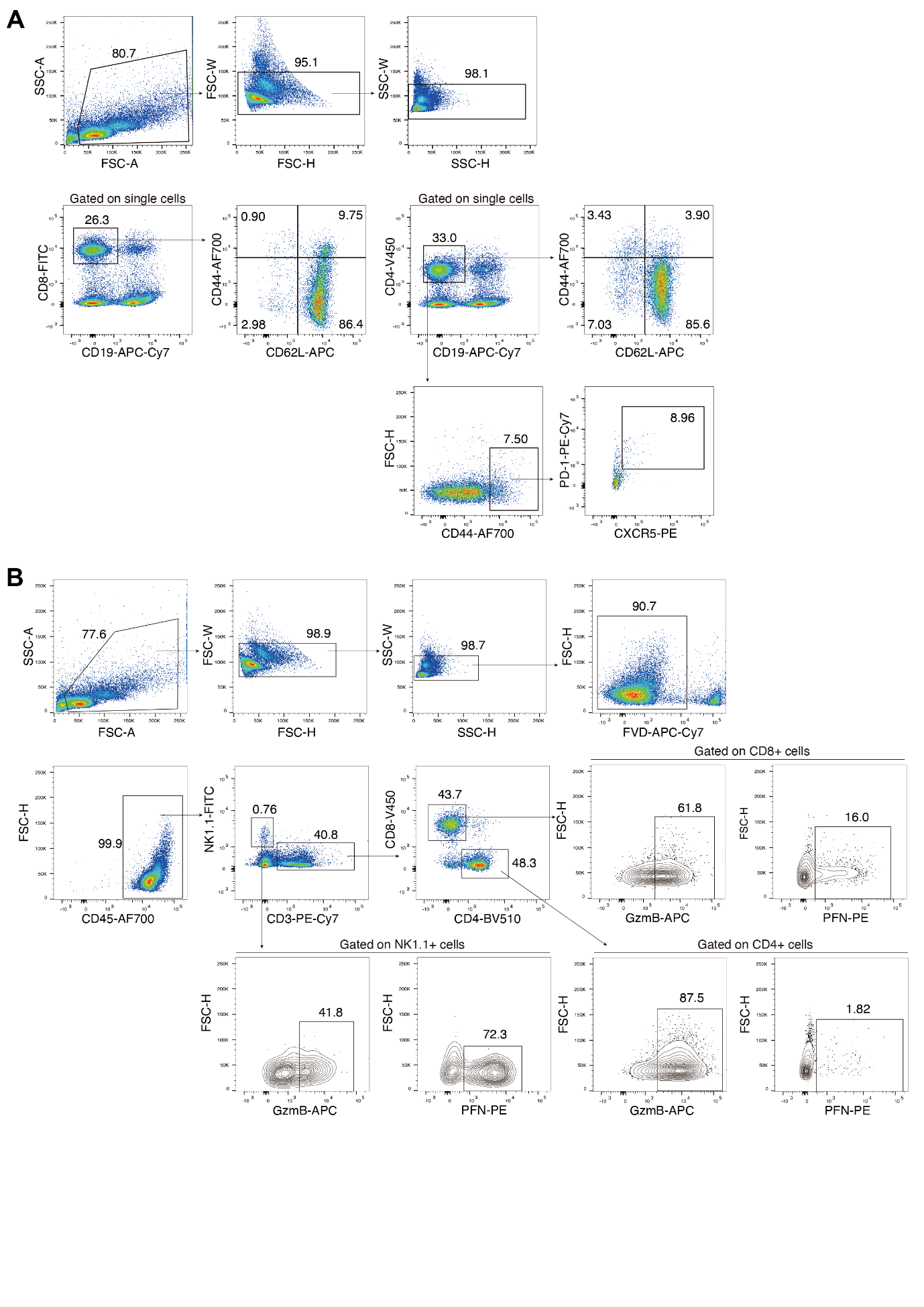


**Figure S9. Gating strategy of cells from lymph nodes**

**
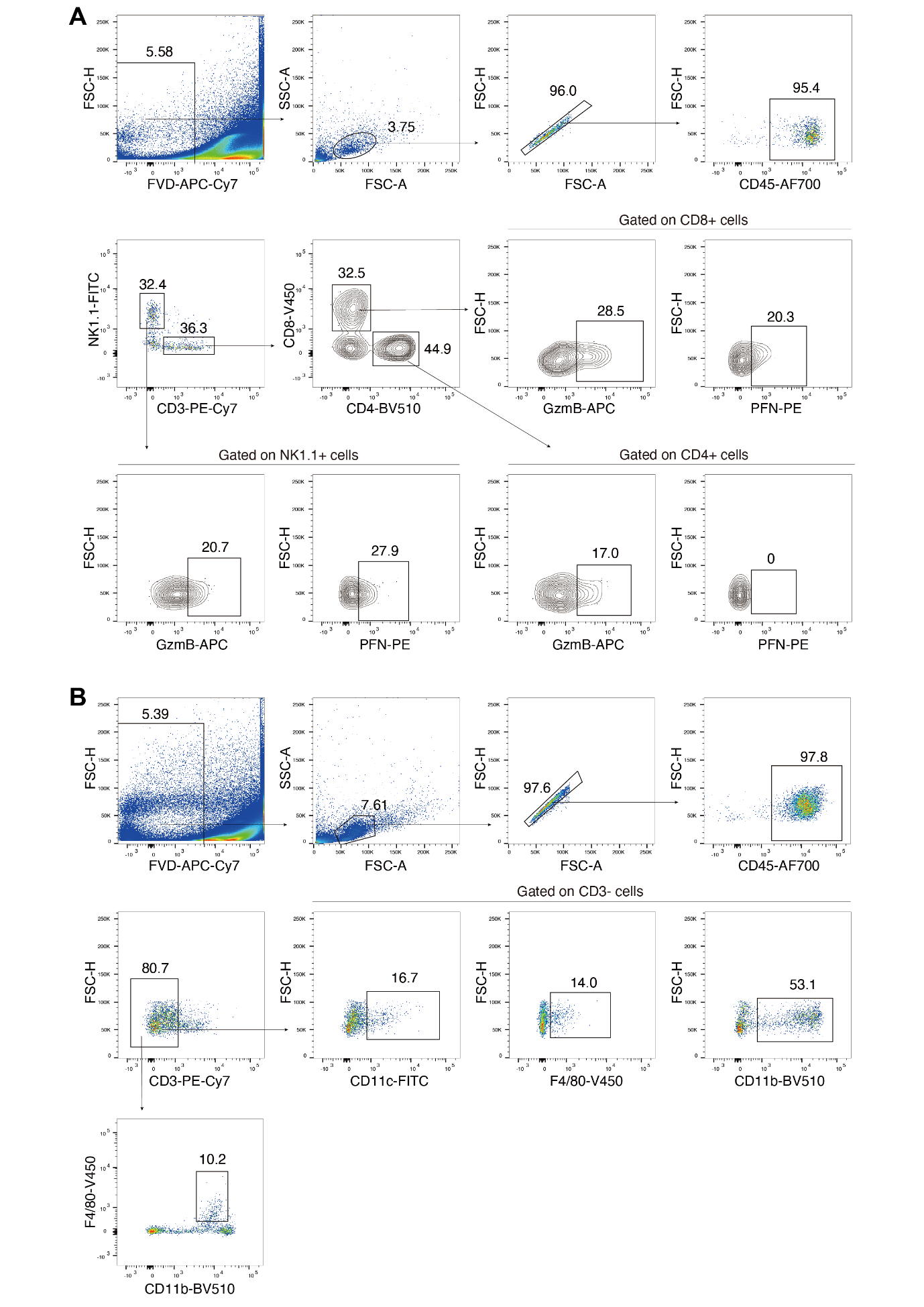
**

**Figure S10. Gating strategy of cells from tumors**

**
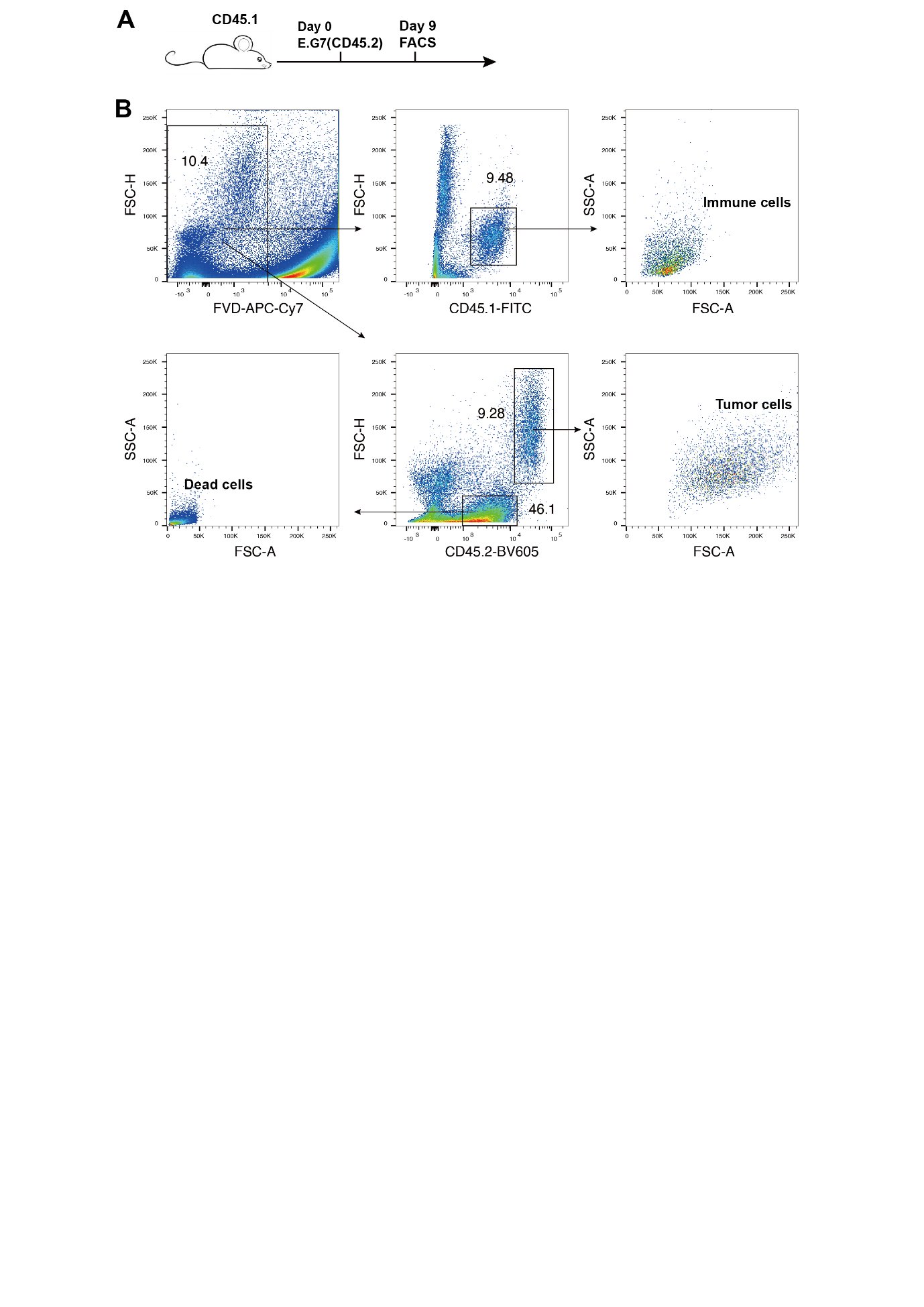
**

**Figure S11. Gating strategy of cells from tumors**

**(A-B)** CD45.1 mice were challenged s.c. with 1 × 10^6^ of E.G7 (CD45.2) cells. On day 9, tumors were removed for flow-cytometry analysis. Immune cells and tumor cells can be distinguished by FSC and SSC.
